# Long-range phase synchronization of high-frequency oscillations in human cortex

**DOI:** 10.1038/s41467-020-18975-8

**Published:** 2020-10-23

**Authors:** G. Arnulfo, S. H. Wang, V. Myrov, B. Toselli, J. Hirvonen, M. M. Fato, L. Nobili, F. Cardinale, A. Rubino, A. Zhigalov, S. Palva, J. M. Palva

**Affiliations:** 1 Neuroscience Center, Helsinki Institute of Life Science, University of Helsinki, Helsinki, Finland; 2 Department of Informatics, Bioengineering, Robotics and System engineering, University of Genoa, Genoa, Italy; 3 Doctoral Programme Brain & Mind, University of Helsinki, Helsinki, Finland; 4 Department of Neuroscience and Biomedical Engineering, Aalto University, Espoo, Finland; 5 Department of Neurosciences, Rehabilitation, Ophthalmology, Genetics and Maternal and Children’s Sciences, University of Genoa, Genoa, Italy; 6 Child Neuropsychiatry Unit, IRCCS Istituto Giannina Gaslini, Genoa, Italy; 7 Centre of Epilepsy Surgery “C. Munari”, Department of Neuroscience, Niguarda Hospital, Milan, Italy; 8 Center for Cognitive Neuroimaging, Institute of Neuroscience and Psychology, University of Glasgow, Glasgow, UK; 9 Present Address: Centre for Human Brain Health, School of Psychology, University of Birmingham, Birmingham, UK

**Keywords:** Neuroscience, Computational neuroscience

## Abstract

Inter-areal synchronization of neuronal oscillations at frequencies below ~100 Hz is a pervasive feature of neuronal activity and is thought to regulate communication in neuronal circuits. In contrast, faster activities and oscillations have been considered to be largely local-circuit-level phenomena without large-scale synchronization between brain regions. We show, using human intracerebral recordings, that 100–400 Hz high-frequency oscillations (HFOs) may be synchronized between widely distributed brain regions. HFO synchronization expresses individual frequency peaks and exhibits reliable connectivity patterns that show stable community structuring. HFO synchronization is also characterized by a laminar profile opposite to that of lower frequencies. Importantly, HFO synchronization is both transiently enhanced and suppressed in separate frequency bands during a response-inhibition task. These findings show that HFO synchronization constitutes a functionally significant form of neuronal spike-timing relationships in brain activity and thus a mesoscopic indication of neuronal communication per se.

## Abstract (web summary)

High-frequency oscillations (HFOs) are common in mammalian brains and have been assumed to be strictly local. Using human intracerebral recordings, the authors find that HFOs can be phase synchronized across long distances between active cortical sites during resting and task states, which may reflect neuronal communication.

## Introduction

Multiple physiologically and morphologically distinct forms of neuronal population activity have been observed at frequencies above 100 Hz. These include broadband high-frequency and high-gamma neural activities (HGA, from 40–150 Hz^1^ to 80–200 Hz^2^), as well as narrow-band fast-gamma oscillations (90–150 Hz)^3,4^, ripple oscillations (140–220 Hz)^3–6^, and fast ripples or fast-electrical oscillations (250–600 Hz)^7^. These oscillatory phenomena have been together termed high-frequency oscillations (HFOs, 80–600 Hz)^3,4^. Sustained HGA and fast-gamma oscillations are a core feature of active cortical processing. HGA is correlated with neuronal firing rates^8^ and the blood-oxygenation-level-dependent signals^9,10^, and is functionally significant in a wide range of perceptual and cognitive processes in rodents^11^, non-human primates^12^, and humans^1,13–15^. Ripple oscillations, on the other hand, have been traditionally associated with hippocampal sharp waves during sleep and off-task states where they are related to memory consolidation^5,16^ and cortex-wide activation^17^, but recent studies show ripple oscillations to play a role in on-task processing, including the retrieval of memories both in humans and rodents^5,17–19^. Pathological HFOs (pHFOs) constitute another group of fast neuronal oscillations in the HFO frequency range and are either mechanistically unique or exacerbated physiological HFOs. pHFOs are characteristic to the epileptic pathophysiology and especially to brain areas underlying seizure generation^16,20–25^.

Phase coupling of oscillatory assemblies, i.e., neuronal synchronization, is ubiquitous in frequencies below 100 Hz and is thought to be instrumental for coordinating neuronal communication and processing^26,27^. Several lines of experimental and theoretical evidence have shown that these phase relations among neuronal oscillations are frequency dependent, decrease as function of neuroanatomical distance, and depend on the axonal conduction delays so that slow oscillations are generally more readily phase coupled over long distances than fast oscillations^28,29^. Accordingly, the apparent absence of inter-areal HGA/HFO synchronization in studies of long-range phase coupling in animal^27,30^ and human^28^ brains is in line with the notion that neuronal oscillations only in frequency bands below 100 Hz exhibit inter-areal phase synchronization.

HGA signals are thought to mainly arise from broadband multi-unit spiking activity^2,31–33^ (MUA) and thus directly reflect the local peri-electrode neuronal population activity per se. Both HGA and MUA are considered to be exclusively local and aperiodic broadband phenomena. Recent studies, however, show that HGA contains both genuine HFO-like oscillatory components and contributions from post-synaptic potentials^34^ that are phase-amplitude coupled with a different phase of theta oscillations than the MUA-correlated HGA. Moreover, in deep layers HGA and MUA are correlated in stimulus-induced responses, but HGA in superficial layers may be observable without MUA, suggesting origins in dendritic potentials^35^. On the other hand, for HFOs, fast post-synaptic currents are central in the generation of the electrophysiological signals because the HFOs are enabled by rhythmicity promoting mechanisms such as network oscillations of fast-spiking interneurons during fast gamma, ripples, and neocortical fast ripples together with gap-junction mediated synchronization^7,36^. Both broadband spiking activity and coherent oscillatory synaptic potentials thus contribute to the genesis of neuronal >100 Hz signals in micro- and meso-scale local-field potential (LFP) signals.

Considering these signal-generation mechanisms and the fact that ripples enable the greatest pyramidal cell synchronization in brain dynamics^3^, we hypothesized that long-range synchronization of HFOs could arise in large-scale networks because local synchronization and high collective firing rates endow local pyramidal cell populations with greatly enhanced efficacy in engaging their post-synaptic targets in remote regions^12,37^. This could be experimentally observable as inter-areal HFO phase coupling and would constitute a direct indication of spiking-based long-range neuronal communication per se.

In animal models and human data, the widespread and long-range impact of ripples has been evidenced by recent observations of pairwise amplitude correlations^38^ and burst co-occurrence of ripple oscillations^3,5,6,18,19^. Ripples bursts co-occur between the rat hippocampus and association cortices during memory formation^19^ so that the hippocampo-neocortical coupling may be mediated by granular retrosplenial cortex^39^. Similar coupling of ripple oscillations is observed between human medial temporal lobe and temporal cortex, and suggested to play a mechanistic role in memory retrieval^18^. However, given the prevalence of phase-amplitude coupling of HGA/HFOs with theta oscillations and the propensity of theta to synchronize across long distances, it remains unclear whether the HFO burst co-occurrences reflect genuine HFO interactions. That is, do burst co-occurrences arise from the impact of ripple-spiking-related post-synaptic potentials in the distant target population, or are the bursts initiated locally via timing mediated by synchronized slow oscillations? Moreover, these findings leave open the possibility of phase correlations in the HFO frequency range. Evidence to this end is provided by a study showing that working memory performance in rats engages high-gamma (~100 Hz) synchronization between hippocampus and entorhinal cortex^11^. Nonetheless, at present there is neither evidence for HFO synchronization in the human brain nor in the large-scale neocortical networks of any species.

In this study, we use an extensive database of resting-state human stereo-electroencephalography (SEEG) recordings to investigate if synchronization in the HFO frequency range (here, 100–450 Hz) would be observable. We use sub-millimeter accurate SEEG-electrode localization^40^ and white-matter referencing^28^ to obtain neocortical meso-scale local-field potential (LFP) signals with little distortion from signal mixing with neighboring gray-matter or distant volume-conducted sources. We find that among these LFPs, long-range HFO synchronization was, in fact, a robust phenomenon limited individually to narrow frequency ranges, and overall stronger than synchronization at around 100 Hz. We rigorously exclude the possibilities of HFO synchronization being attributable to putative confounders such as the epileptiform pathophysiology or physiological and technical artefacts. We find HFO synchronization to exhibit split-cohort reliable large-scale connectivity structure and a laminar profile that was distinct from those of lower frequencies. We further find that HFO synchronization was dynamically strengthened among task-relevant cortical areas in a visuomotor task. These findings thus demonstrate for the first time that HFO synchronization characterizes human brain activity and comprises a functionally significant form of spatio-temporally highly accurate neuronal coupling therein.

## Results

### Probing human large-scale brain dynamics with SEEG

**Fig. 1:**
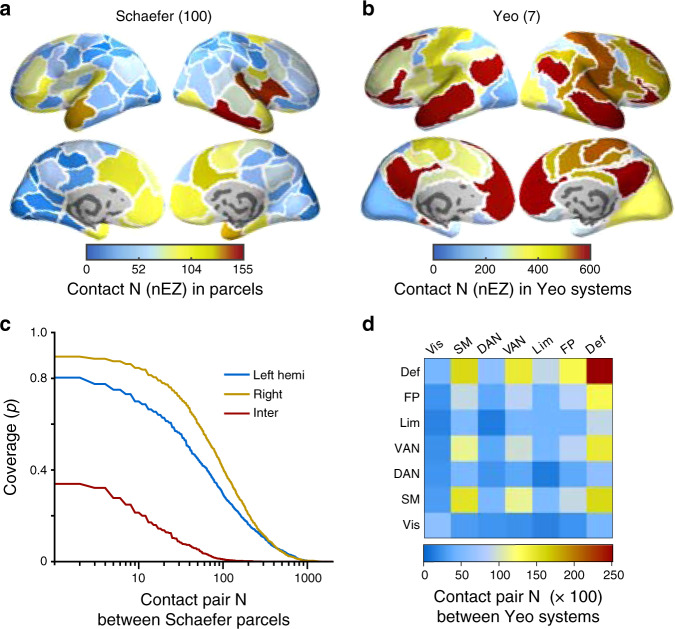
Anatomical sampling statistics and coverage for assessment of cortical interactions. **a** Numbers of SEEG electrode contacts in the non-epileptogenic zone (nEZ) across 100 parcels (Schaefer) and **b** seven functional systems (Yeo). **c** Population level cortical interaction coverage in terms of the total number of SEEG contact pairs in Schaefer parcellation within the left and right hemispheres, and between them (Inter), and **d** among the Yeo functional systems (Vis visual, SM somatomotor, DAN dorsal attention, VAN ventral attention, Lim limbic, FP fronto-parietal, Def default, both hemispheres pooled).

We recorded ~10 min of undisturbed resting-state human intracerebral local-field potential (LFP) signals from 92 consecutive patients using stereo-electroencephalography (SEEG). From this cohort, 25 patients were later excluded either because of previous brain surgery, such as temporal lobotomy, or cortical malformations identified in anatomical MRI (Supplementary Table 1). After exclusion of electrode contacts in the epileptic zone (EZ), the final cohort of 67 patients yielded a total of 7068 gray matter contacts (113 ± 16.2 per subject, mean ± SD, range 70–152) that gave a dense sampling across all neocortical regions (Fig. 1a) and of seven canonical functional brain systems defined by functional magnetic resonance imaging (fMRI) intrinsic connectivity mapping^41,42^ (Fig. 1b, more details of sampling statistics see Supplementary Fig. 1).

In this study, we assessed the phase interactions between all SEEG electrode contacts that did not share a reference contact and were located in neocortical gray-matter outside of the EZ (nEZ, 5500 ± 1600 (mean ± SD) contact pairs per subject, range: 2094–9947, a total of 368,043 contact pairs). Out of all possible within-hemispheric connections in the 100-parcel atlas, this sampling yielded ~80% of the left- and ~90% of the right-hemispheric connections (Fig. 1c) and provided abundant sampling at the level of seven functional systems (Fig. 1d). The present data can thus be used to obtain comprehensive insight into large-scale brain dynamics and connectivity.

### HFOs exhibit inter-areal synchronization

**Fig. 2:**
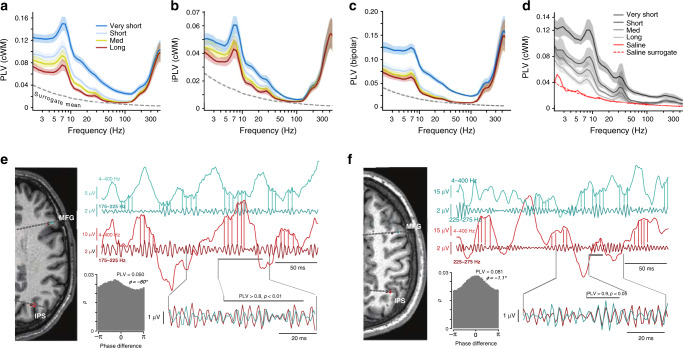
Long-range synchronization of high-frequency oscillations. **a** Mean phase synchronization, as measured with the phase-locking value (PLV) and **b** imaginary PLV (iPLV) between all pairs of cWM-referenced SEEG contacts in distance-range quartiles (92,011 contact pairs in each quartile; very-short: < 32 mm; short: 32–45 mm; medium: 45–60 mm; long: 60–137 mm. Dashed lines are the 99.9th%-ile of surrogate data (*N*_randomizations_ = 100). Shaded areas are the 2.5–97.5%-ile bootstrap confidence-limits of the mean PLV/iPLV (*N*_bootstraps_ = 100)). **c** Mean phase synchronization among bipolar-referenced SEEG contacts. **d** Phase synchronization between SEEG contacts in saline solution (red lines, synchronization spectra from **a** are shown in gray for comparison). **e** Examples of significant (*p* < 0.001) long-range HFO synchronization with non-zero and **f** near-zero phase lags (*ϕ*) from two subjects (MFG middle frontal gyrus, azure traces and gray-matter contacts in MRI insets, IPS intra-parietal sulcus, red; black circles in MRI insets mark the white-matter reference channels).

**Fig. 3:**
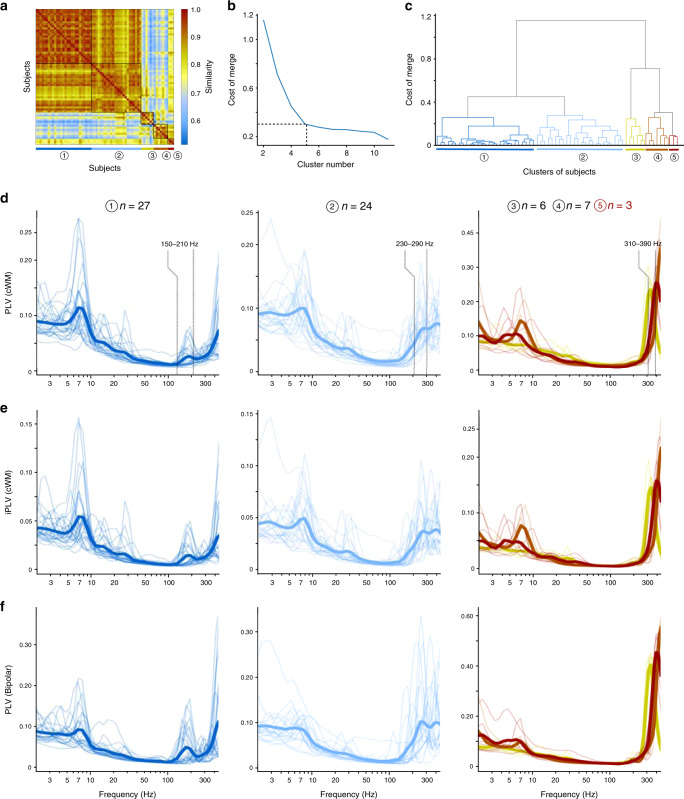
Clustering subjects by similarity in synchrony patterns across frequencies. **a** Similarity matrix of HFO synchronization profiles (cWM) across the subject cohort. The matrix was sorted by hierarchical clustering and partitioned to five clusters (black squares corresponding to dendrogram in **c**). **b** The cost of merging subject clusters. **c** The dendrogram derived from the similarity matrix in **a** showing the individual subjects and their division into the five subject clusters. **d** Individual (thin lines) and mean (thick lines) cWM- referenced PLV, **e** iPLV, and **f** bipolar-referenced PLV of the subject clusters.

We estimated inter-areal phase correlations first by Morlet-wavelet filtering the data into narrow-band time series (50 frequencies from 2 to 450 Hz) and then by using the phase-locking value (PLV) to quantify inter-areal phase synchronization among all nEZ SEEG contacts (“Methods”). To obtain polarity-correct LFP recordings with controlled signal mixing and volume conduction effects (Supplementary Methods), we re-referenced the gray-matter electrode contacts to the closest ones in white-matter (cWM)^28^. We recently demonstrated that this referencing approach performs better than classical bipolar (i.e., referencing to neighboring channel) in terms of signal mixing and polarity consistency (Supplementary Notes). The PLVs were first averaged across subjects in four quartiles of inter-contact distances (Fig. 2a). In all distance quartiles, the mean PLV increased from 2 to 7 Hz and then decayed from 10 to 100 Hz, as found earlier^28^. Throughout the 100–450 Hz HFO frequency band, however, inter-areal synchronization exhibited peaks at around 150–210 Hz and 300–400 Hz (Fig. 2a). To test whether this result could be confounded by residual volume conduction, we used imaginary PLV (iPLV) to compute inter-areal synchronization but found essentially the same result, which indicates that synchronization is not artificially inflated by volume conduction^43^ (Fig. 2b). The same phase synchronization profile was also observed by using bipolar referencing that is another strict control over volume conduction (Fig. 2c and Supplementary Fig. 1g). Note that although the mean PLV of very-short distance contact pairs (0–32 mm) is shown in Fig. 2, the PLV estimates in this distance range may be inflated by residual volume conduction. To then assess the extent of HFO synchronization in brain-wide cortical networks, we evaluated the fractions of significant PLV estimates (PLV values greater than surrogate PLV at *p* < 0.001) and found that even at distances >60 mm, HFO synchronization was significant in >50% of contact pairs (Supplementary Fig. 1h). The HFO synchronization was also reliably observed with randomly split cohorts with matched anatomical sampling indicating essentially that in these data, the result can be observed twice at half of the cohort size (Supplementary Fig. 2a) with a high correlation between the split cohorts (Supplementary Fig. 2b). To obtain an additional, independent line of corroborating evidence, we performed the HFO synchronization analyses to a publicly available set of electrocorticography (ECoG) data (Supplementary Notes). These subdural ECoG data revealed HFO synchronization with peaks at 200–300 Hz (Supplementary Fig. 2j–m) that was similar to our SEEG data (Fig. 3). HFO synchronization thus appears to be a reliable and widespread phenomenon in intracerebral recordings of human resting-state brain activity.

To corroborate this, we asked if environmental noise or amplifier noise correlations could constitute a technical confounder. We acquired 10 min of data with two SEEG electrode shafts (18 contacts each) immersed in saline solution. Performing the same analysis pipeline as above with bipolar re-referencing revealed no evidence for either <100 Hz or HFO phase synchronization above the noise level estimated with surrogate data (Fig. 2c, red line).

To check that HFO synchronization did not arise as filtering artefacts from spikes, we first compared visually broad- and narrow-band filtered time series (Fig. 2e, f). These data revealed short bursts of significant HFO coupling over centimeter-scale distances. Notably, transient narrow-band HFO synchronization was observed as low-amplitude fluctuations that were visible in the broadband time series, which provided initial evidence for that HFO synchronization was not attributable to spikes or technical artefacts.

Given the novelty of these observations, we performed a series of control analyses to exclude the possibilities that HFO synchronization arose artefactually from technical or physiological sources. To extend the control analyses beyond volume conduction and amplifier noise (see Fig. 2 and Supplementary Figs. 1–3), we tested whether HFO synchronization could be attributable to (i) epileptiform neuronal activity, such as interictal spikes (Supplementary Fig. 3d), and (ii) muscular signals (Supplementary Fig. 3b), (iii) leakage of line noise and notch-filter artefacts (Supplementary Fig. 4), and (iv) effects of filtering near the Nyquist frequency (Supplementary Fig. 5). The results of these analyses converged to show that HFO synchronization appears only explainable by true correlations between HFO signals from distant neuronal assemblies (Supplementary Notes). This conclusion was further consolidated by the findings, as detailed below, that HFO synchronization had high inter-individual variability, was predominant in specific functional brain systems, had a community structure and laminar connectivity profile that were distinct from those of slower activities, and showed narrow-band task dynamics, which together appear inconceivable for technical or physiological artefacts.

### HFO synchronization spectra show individual peaks

To investigate inter-individual variability in the synchronization profiles and to test for the presence of outliers, we quantified the similarity of individual HFO-range PLV spectra for cWM-referenced data across all subjects (Fig. 3a, EZ contacts and very-short distances excluded). Hierarchical clustering with optimal solution defined by merging cost (Fig. 3b) demarcated this similarity matrix into five clusters of subjects. The corresponding dendrogram showed that the first two clusters accounted for 51 out of 67 subjects (Fig. 3c). In these clusters, 1 and 2, the subjects exhibited HFO synchronization in separate peaks at around 150–210 Hz and 210–300 Hz, respectively (Fig. 3d). These peaks were also clearly visible in analyses performed with iPLV (Fig. 3e) and bipolar referencing (Fig. 3f). The clusters 3 and 4 contained 13 subjects together and were characterized by a sharp peak in the synchronization spectra at 300–400 Hz and by the lack of the 150–300 Hz components visible in clusters 1 and 2. The three subjects in the fifth cluster exhibited only a ramp-up of synchronization near the Nyquist limit (500 Hz) and were excluded from further analysis because the lack of a peak implied that the 1 kHz SEEG sampling rate was inadequate for this cluster (Supplementary Fig. 5). These findings exclude the possibility that these long-range synchronization phenomena relate to broadband HGA signals. Instead, the observed organization in narrow frequency bands directly implicates HFOs and indicates that oscillatory mechanisms regulate the timing of coherent spiking output of the underlying local assemblies.

### Anatomical architecture of HFO synchronization

**Fig. 4:**
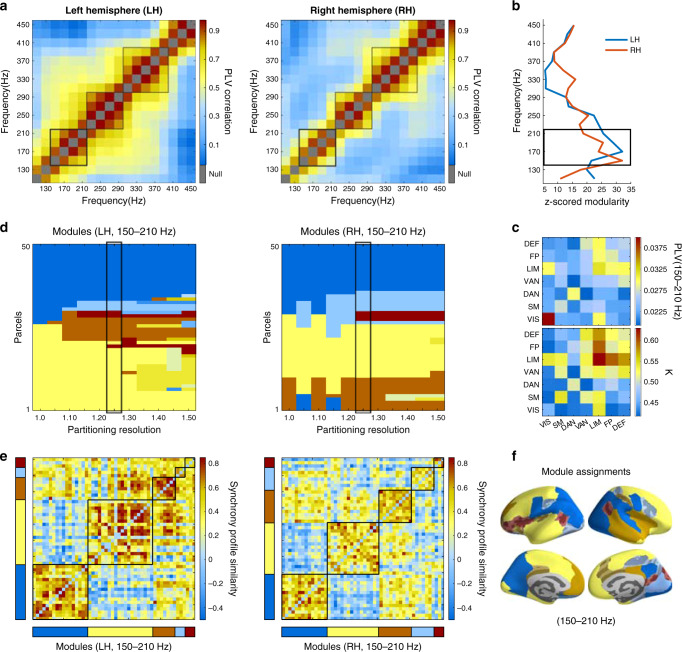
Neuroanatomical localization and community structures in HFO synchrony networks. **a** Topological similarity between narrow-band PLV connectomes in Schaefer 100-parcel parcellation. **b** Mean normalized modularity (z-score against degree-distribution matched surrogate networks) assessed with Leiden community detection (see Supplementary Fig. 5). **c** Mean contact-pair PLV and fractions of significants (*K*) between the seven functional systems of Yeo parcellation in the 150–210 Hz frequency band. **d** Community identities of cortical parcels as a function of the resolution parameter, *γ*, of the Leiden community detection algorithm. **e** Communities at *γ* = 1.25 (black squares) in sorted synchronization similarity matrices. **f** Communities of (**e**) illustrated on inflated cortical surfaces (parcels with significantly stable community assignment rendered opaque (one-tail test, *p* < 0.05, parcel module-allocation stability, Supplementary Methods)). Module assignments to 22% left and 14% right-hemispheric parcels were unstable one-tail test, (*p* > 0.05, parcel module-allocation stability, Supplementary Methods) and rendered semi-transparent.

To address the neuroanatomical characteristics of HFO synchrony at the population level, we used PLV connectomes where cWM-referenced SEEG contact pairs were pooled into Schaefer parcel pairs across the subjects in clusters 1 and 2 (*n* = 51, see Supplementary Methods) that had high mutual similarity among their PLV spectra (see Fig. 3a). We studied the left and right hemispheres separately because the interhemispheric connections were inadequately sampled (see Fig. 1c). We first asked how similar the connectomes of HFO synchrony were across frequencies. The connectome similarity was high only in limited clusters of wavelet frequencies (Fig. 4a) that were found in the same frequencies as the peaks in PLV spectra in the three bands, roughly 150–210 Hz, 210–300 Hz, and 300–400 Hz. These findings further support the notion that HFO synchronization was indeed a narrow-band phenomenon with distinct cortical coupling networks in different frequency bands. Conversely, these data reject the alternative hypothesis: if HFO synchronization were attributable to broadband MUA-like HGA signals, these similarity matrices would have shown widespread correlations between frequencies. Corroborating this result, we found essentially the same frequency clusters by estimating the cross-frequency similarity of network node (parcel) strengths (Supplementary Fig. 6a–c).

We then addressed the anatomical structuring of the HFO connectomes. HFO synchronization networks exhibited modular structures with significant normalized modularity (*z*-scores > 5) throughout the HFO frequency range (Fig. 4b), and the modularity peaked in both hemispheres in the 150–210 Hz band (Fig. 4b and Supplementary Fig. 6d). To assess the anatomical patterns of 150–210 Hz synchronization, we first pooled the nEZ electrode contact pairs in the seven functional system Yeo parcellation. At this coarse spatial resolution, inter-system HFO synchronization was conspicuously inhomogeneous between the functional systems^41^ and dominated by the coupling within the limbic system and between the limbic and other systems (Fig. 4c; for other HFO bands, see Supplementary Fig. 2g–i). This anatomical pattern was reproducible with split cohorts and highly correlated between the splits (Supplementary Fig. 2c, d). At a finer anatomical resolution of the 100-parcel Schaefer parcellation, we then estimated the similarity of 150–210 Hz HFO synchrony patterns among parcels and used Leiden community detection to identify modules in this matrix to group parcels that have similar connectivity with the rest of the network (for inter-parcel PLV adjacency matrices and nodal strengths, see Supplementary Fig. 6e, f). We evaluated community structures across a range resolution parameter values^44^ *γ* = 1–1.5 and observed that the HFO synchronization networks were progressively split into 2–11 and 2–7 modules in the left and right hemispheres, respectively (Fig. 4d). Visualization of the five modules at *γ* = 1.25 showed that the synchrony similarity matrix was primarily partitioned into two main modules (Fig. 4e), that similarly in the left and right hemispheres corresponded to posterior (Fig. 4f, blue and light blue) and fronto-central brain areas (yellow and orange). These broad divisions where then further split into occipital and temporal modules. Overall, these findings suggest that HFO synchronization has a coarse, bilaterally symmetric anatomical community structure that follows the anterior-posterior gradient earlier observed in both structural and functional networks and gray matter thickness^45^. HFO synchronization thus has a group-level stable cortical topology that is robust against individual subjects and individual pathogenesis or electrode placement. We cannot, however, rule out the possibility that there may be aspects in cortical localization of epilepsy, electrode placement, and selection of subjects into pre-operative SEEG, which are consistent across subsets of subjects.

### Laminar profile of HFO synchronization

**Fig. 5:**
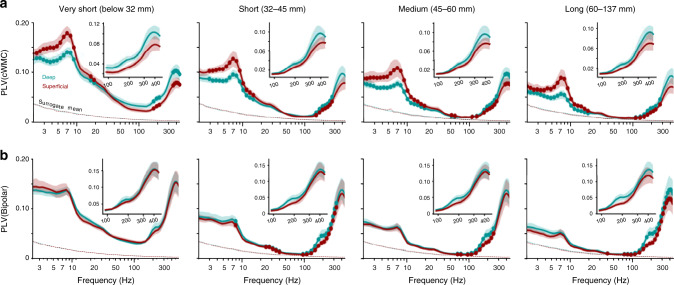
HFO synchrony has a laminar profile opposite to that of low-frequency synchrony. **a** PLV spectra for contact pairs located in deep (−0.3 < GMPI ≤ 0; teal) and superficial cortical layers (0.5 < GMPI < 1; red) with cWM and **b** bipolar reference (GMPI indicates the normalized depth of the electrode contact in gray matter so that GMPI = 0: white/gray-matter surface and GMPI = 1: pial surface, see Supplementary Fig. 2). The markers indicate significant differences (two-tailed permutation test, *N*_permutations_ = 100: *p* < 0.05, Benjamini–Hochberg method at *α* < 0.05). Inset: mean PLV in HGA frequencies. Dashed lines indicate the 99.9th%-ile of the surrogate data (*N*_randomizations_ = 100). Shaded areas indicate the 2.5–97.5%-ile bootstrap confidence-limits of the mean PLV (*N*_bootstraps_ = 100).

Deep and superficial cortical laminae contribute differently to inter-areal phase-synchronization at frequencies below 100 Hz^28^. We next asked whether HFO synchronization networks would show similar differentiation between the cortical laminae. Leveraging the accurate localization of the SEEG contacts, we divided the electrode contacts into deep and superficial by their relative position in cortical gray matter between the white-matter and pial surfaces (“Methods”). We assessed synchronization between the deep-deep and superficial–superficial contacts pairs with PLV (Fig. 5) and iPLV (Supplementary Fig. 7) using both cWM and bipolar referencing. In the cWM-referenced data, replicating our prior observations^28^, we found the superficial contact pairs to show stronger synchronization than the deep contact pairs across all distance ranges in the 3–20 Hz frequency range. This laminar difference in the theta- and alpha-frequency range synchronization is in line with the laminar localization of the current sources underlying theta and alpha oscillations in macaques and humans^26,46^. In contrast, HFO synchronization in cWM-referenced data was stronger (Fig. 5a) and more prevalent (Supplementary Fig. 7) among signals from deep cortical layers in all distance quartiles (*p* < 0.05, Benjamini–Hochberg method at *α* < 0.05). This pattern was split-cohort reliable (Supplementary Fig. 2e–f) and was also observed with bipolar referencing (Fig. 5b) and iPLV (Supplementary Fig. 7b). Importantly, the laminar separation was predominantly observed in the 150–300 Hz band, either alone or as a separate cluster from higher frequencies (Fig. 5), further supporting the notion of separable physiological narrow-band processes underlying HFO synchronization. Interestingly, the laminar difference in low frequencies was abolished in bipolar referencing while both the laminar HFO synchronization difference and HFO PLV values were enhanced. This suggests that the HFO signals arise in very local assemblies in the peri-electrode volume while the low-frequency signals reflect more widespread assemblies and much greater volume conduction. These results thus indicate that long-range synchronized HFOs at least partly originates from current sources distinct from those underlying the slower oscillations.

### HFO synchronization is associated with high-amplitude HFO

To further assess the physiological plausibility of HFO synchronization, we asked whether it was related to the moment-to-moment variability in the amplitudes of local HFO. HGA and HFO amplitudes reflect primarily the coherence of local neuronal MUA or of the post-synaptic potentials^33^ in populations of at least hundreds of neurons in the immediate vicinity of the electrode contact that may pick signals from up to ~50 k neurons within a range of ~100 μm. As coherent neuronal firing is essential for a local assembly to engage its post-synaptic targets effectively, we hypothesized that moments of strongest HFO synchrony would be associated exclusively with the presence of high-amplitude HFO in both electrode contacts of the pair. We selected electrode contacts exhibiting significant (*p* < 0.001) HFO synchronization and for each contact-pair, distributed the data sample-by-sample into a two-dimensional (2D) matrix according to HFO amplitude quintiles of the contacts (see Methods).

**Fig. 6:**
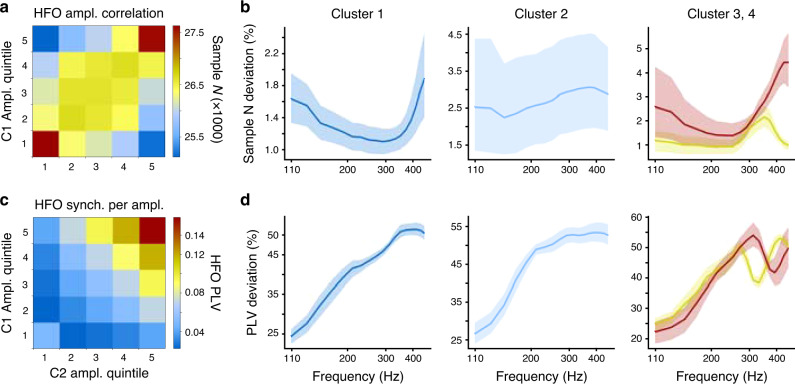
HFO synchrony is the strongest during moments of high-amplitude HFO. **a** Histogram of the HFO amplitude coincidences for all pairs of contacts (C1 and C2), showing a saddle with peaks when both amplitudes are either at their highest or lowest values. **b** Absolute mean deviation (%) of numbers of samples in **a** from uniform distribution as a function of high-gamma frequency. Shaded areas indicate bootstrap confidence intervals for the mean (5 and 95%-tile, *N*_bootstraps_ = 1000)). **c** Moment-to-moment HFO synchronization (PLV) for SEEG electrode contact pairs (C1, C2) is dependent on the HFO amplitude at both contacts. Each matrix element is the mean of instantaneous PLV between all significant contact pairs as a function of their moment-to-moment normalized amplitudes. **d** Mean absolute deviation (%) of the observed PLV in bins of contact amplitudes (see **c**) from uniform distribution as a function of high-gamma frequency. Shaded areas indicate bootstrap confidence intervals for the mean (5 and 95%-tile, *N*_bootstraps_ = 1000).

First, inspecting the variability in the numbers of samples among the cells of this 2D matrices (Fig. 6a) we found that there was a slight positive correlation between the amplitudes so that the coincidence of amplitudes in the largest quintiles was ~8% more prevalent (i.e., 27.5/25.5 − 1) than the coincidence of the smallest and largest amplitudes (Fig. 6a). This amplitude covariance indicates the presence of weak inter-areal HFO amplitude correlations. To elucidate how the amplitude correlation depended on frequency, we plotted the absolute mean difference of the observed distribution from the null hypothesis. The amplitude correlations were salient at all frequencies but confined to a total deviation of 1–5% from the null hypothesis level (Fig. 6b). We then estimated the inter-contact PLVs in the amplitude quintile bins with equalized sample counts and averaged the PLVs across electrode pairs, subjects, and frequencies. We found that HFO phase coupling was indeed the strongest in those moments when the HFO amplitudes were the largest in both contacts and much weaker when either location exhibited the lowest HFO amplitudes (Fig. 5c). In contrast with the ~8% max effect of the amplitude correlations, the difference between smallest (~0.03) and largest PLV (~0.15) observations was ~500%. The mean deviation of the PLV values across quintiles from the null hypothesis of no amplitude dependence ranged from 25 to 55% with a frequency dependence that matched the subject-cluster PLV profiles (Fig. 5d, see Fig. 3d). To exclude the possibility that changes in the signal-to-noise ratio (SNR) could underlie this finding, we used saline recordings to estimate the apparent SNR and compared these values against our earlier study on how SNR influences PLV estimates. We found that, given the SNR range of HFOs in these data (4–10), the mean PLV (~0.06) may be inflated only by ~10% by any amplitude increase (see Supplementary Notes and Fig. 3), i.e., an order of magnitude less than observed here. These data thus clearly show that high-amplitude local HFO, assumed to largely reflect high local HFO coherence^33^, is instrumental for long-range HFO synchronization.

### Phase-amplitude coupling of HFO and slow oscillations

**Fig. 7:**
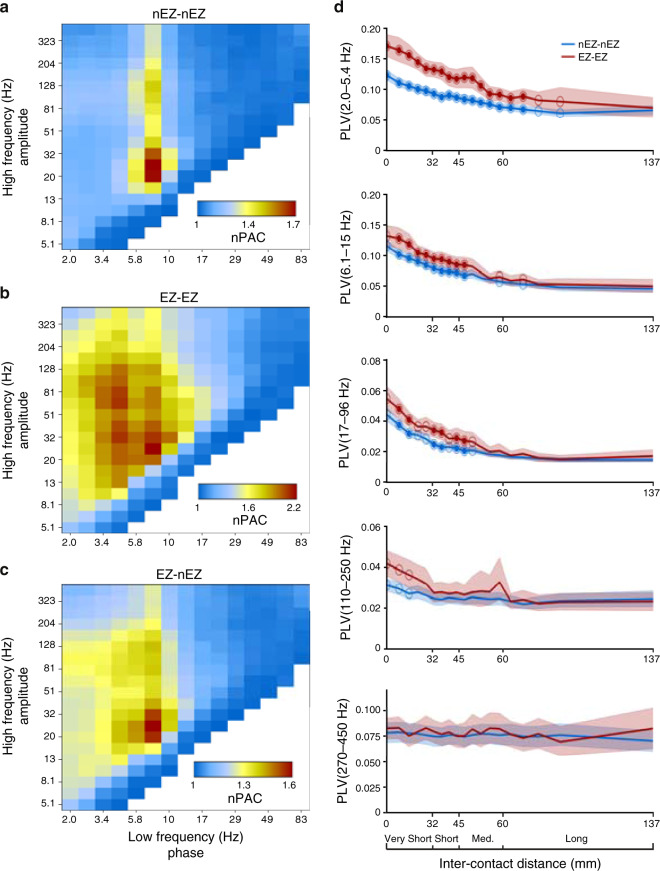
Abnormal HFO PAC and aberrant synchrony below 100 Hz characterize EZ contacts. **a** Cross-frequency phase-amplitude coupling (PAC) among SEEG contacts outside of the epileptogenic zone (nEZ–nEZ). PAC was evaluated with PLV_PAC_ and here illustrated as normalized PAC, nPAC = PLV_PAC,observed_/PLV_PAC,surrogate_, so that nPAC > 1 indicates PAC above the null hypothesis level. Extent of coupling measured as the fraction of significant edges exhibiting PAC (*K*) is reported in Supplementary Fig. 3c. **b** PAC among contacts in the epileptogenic zone (EZ–EZ) and **c** between the putatively healthy and epileptogenic contacts (EZ–nEZ). **d** Synchronization among EZ–EZ (red) and nEZ–nEZ (blue) contact pairs in 20 bins of inter-contact distances for frequency ranges from delta to HGA (top to bottom panels). Shaded areas indicate the 5 and 95%-ile of mean PLV of bootstrap observations (*N*_bootstraps_ = 10^4^, markers denote the frequencies with a significant (*p* < 0.05, one-sided randomization test with Benjamini–Hochberg correction) difference between EZ–EZ and nEZ–nEZ PLV values, solid makers denote differences that are significant after Benjamini–Hochberg FDR correction for multiple comparison).

The nesting of fast oscillations in cycles of slower oscillations is an often-observed phenomenon in electrophysiological recordings at both <1 Hz^47,48^ and >1 Hz frequencies^34,49^. We assessed whether local HFO narrow-band amplitudes were nested within specific phases of slower oscillations by evaluating phase-amplitude coupling (PAC, “Methods”) among all contact pairs and frequencies. We also aimed to dissociate healthy from pathological PAC and thus included data from both the putatively healthy brain areas (nEZ) and EZ. Among the nEZ electrode contacts, beta- and low-gamma (20–40 Hz) oscillation amplitudes were strongly coupled with the phase of theta- to alpha-band oscillations (5–10 Hz, peaking at ~8 Hz) (Fig. 7a). The amplitude of HFOs, with PAC peaking in the 100–200 Hz band, was coupled with the same 5–10 Hz oscillations. However, in EZ, PAC was much more widespread. In addition to the ~8 Hz low-frequency and ~130 Hz high-frequency peaks, PAC in EZ was characterized by a low-frequency peak in the delta-frequency band (1–4 Hz) and a gamma-band peak at ~80 Hz (Fig. 7b). Reflections towards these putatively pathological delta-band PAC components were observed also in PAC between EZ and nEZ contact pairs (Fig. 7c).

### HFO synchronization is a characteristic of healthy brain activity

We next asked whether HFO synchrony is a feature of putatively healthy brain activity or a byproduct of the epileptic pathogenesis. The latter hypothesis would be supported by aberrant HFO synchronization within the epileptogenic network. We addressed this question by asking whether HFO synchronization was significantly stronger between electrode contacts in the EZ than between those in nEZ. Controlling for the between-contact neuroanatomical distance, we found that the EZ–EZ contact pairs indeed exhibited stronger synchronization than nEZ–nEZ contact pairs but only in frequencies up to 96 Hz (Fig. 7d). We found no significant differences in the HFO frequencies across all distances with false discovery rate (FDR) corrected statistics (*p* > 0.05, randomization test, Benjamini–Hochberg FDR correction, see Fig. 7d). We further tested whether the strength of HFO synchronization was correlated with the frequency of interictal spikes that constitutes a proxy for the severity of epileptic pathology, but no such correlation was observed (Supplementary Fig. 3d). These data thus suggest that HFO synchronization is predominantly a property of healthy brain dynamics.

### HFO synchronization is transiently enhanced in a visuomotor task

**Fig. 8:**
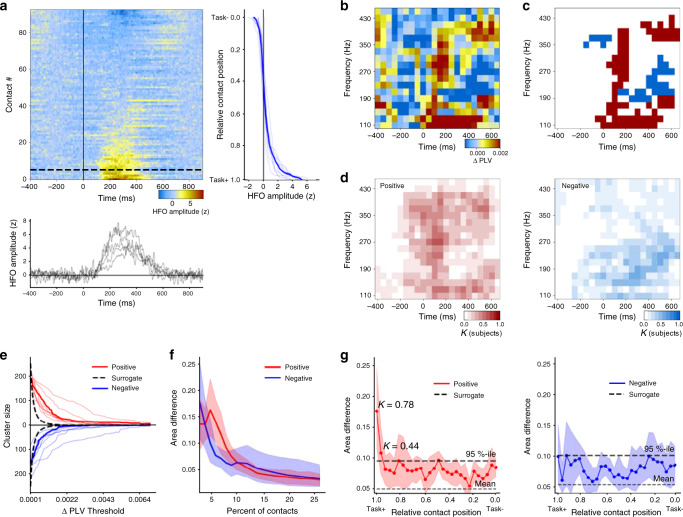
Transiently enhanced HFO synchrony among task-relevant areas during visuomotor processing. **a** HFO amplitude is increased above baseline levels in a subset of SEEG electrode contacts (representative subject, contacts sorted by the mean HFO amplitude in 150–350 ms time window, frequencies averaged across 110–430 Hz, *z*-score normalized by the pre-stimulus baseline from –500 ms to –10 ms). The vertical line plot indicates the mean (thick line) and individual (thin lines) sorted HFO responses for all subjects in the 150–350 ms window. The horizontal line plot shows the mean (thick line) and individual (thin lines) temporal evolution of the HFO responses for the top 5% of electrode contacts for all subjects. **b** Change in PLV from baseline levels among the top 5% of electrode contacts (200 ms time windows for frequencies from 110 to 450 Hz frequencies for the same subject that is shown in **a**). **c** Three largest positive (red) and negative (blue) time-frequency clusters for the representative subject (threshold for PLV change: 0.001). **d** Superposition of the three largest positive (left) and negative (right) clusters of all subjects (*K* indicates the fraction of subjects with a cluster contributing to each time-frequency element). **e** Sum of the time-frequency elements in the three largest clusters as a function of the clustering threshold for the positive and negative clusters (thick lines, group mean; thin lines, individual subjects; black dashed line indicates 95%-ile of the cluster sizes found with identical analysis of surrogate data). **f** Difference of total cluster-size areas (area-under curves in **e** from threshold of 0 to inf) from threshold between the data and surrogate mean for varying fractions of contacts used in synchrony estimation (averaged across subjects, shaded areas indicate bootstrapped 5 and 95 %-ile confidence limits of the means). **g** Cluster-size areas (data minus surrogate as in **f**) for synchronization evaluated in 5% contact sets along the Task+ … Task– axis defined by sorted HFO amplitudes (black lines indicate the 95%-ile (thick line) and mean (thin line) of surrogate data.

To investigate whether the HFO synchronization observed during awake rest was dynamically modulated during task performance, and thereby potentially functionally relevant, we inspected time-resolved phase synchronization when patients performed a visuomotor Go/No-Go task. We acquired task-state SEEG data from an additional cohort of 11 patients who performed a visual Go/NoGo response-inhibition task where they reacted with a button press to Go stimuli (blue rectangles, 75% probability) and withheld responses to rare NoGo stimuli (yellow rectangles, 25% probability). In this task, response-inhibition is known to involve large-scale fronto-parietal brain activation. We first examined the peri-stimulus amplitude dynamics of local HFOs that is known to localize task-relevant brain regions with high accuracy^3,5–7,18,19,39^. We found the mean HFO amplitude during 150–350 ms to increase above two baseline SDs in nine subjects in 4–11% of their 103–139 gray matter recording sites (Fig. 8a). Two subjects showed no HFO amplitude responses, presumably because of technical reasons, and were excluded from further analyses. We examined HFO synchronization among the 5% subset of cortical regions that were most task-relevant (Task+, see Fig. 8a) in terms of exhibiting the greatest task-induced HFO amplitude effects. Time-frequency representations of changes in HFO synchronization from baseline levels revealed time-frequency clusters of both strengthened synchronization and desynchronization (Fig. 8b). We applied cluster permutation statistics to identify these clusters in individual subjects (Fig. 8c). First, pooling the three largest clusters across subjects shows transient wideband HFO synchronization that peaks during the first 200 ms after stimulus presentation and both sustained narrow-band HFO synchronization in 110–150 and 300–400 Hz bands (Fig. 8d left) and concurrent HFO desynchronization between 150 and 250 Hz (Fig. 8d right). To ensure that these findings were robust against the choice of thresholds, we quantified the total size of the three largest clusters across all thresholds and compared this with cluster sizes obtained from surrogate data with an identical analysis. All subjects exhibited cluster sizes greater than the 95 %-iles of surrogate data across a wide range of cluster PLV thresholds (Fig. 8e). We then asked in how widespread brain regions was this HFO synchronization observable. To obtain a robust and threshold-independent measure of the HFO dynamics, we evaluated the difference in cluster-size areas of real and surrogate cluster sizes across all thresholds (see Fig. 8e) separately for positive and negative clusters. This area difference was largest for sets of contacts that were below 10% of all contacts (Fig. 8f), which matches well with the numbers of most robustly task-relevant contacts in HFO amplitude data (see Fig. 8a). Finally, to confirm that this transient HFO synchronization was localized to the task-relevant brain areas, we evaluated the area difference for all 5% contact sets along the Task+… Task– axis of sorted HFO amplitudes (see Fig. 8a). This analysis revealed significant positive HFO synchronization clusters only in the first two locations (first and second 5%, Fig. 8g) thus confirming that HFO synchronization was strictly limited to task-relevant circuitry and also that it was not artificially inflated by the analysis procedure. In single-subject statistics, 7/9 (78%) of subjects exhibited significant positive effect (see Fig. 8g).

These data thus show that HFO synchronization is strengthened transiently during task-relevant neuronal processing specifically in the task-positive cortical areas. We suggest that HFO synchronization in these circuits reflect the neuronal communication per se underlying large-scale coordination of visuomotor processing in the Go/NoGo task.

## Discussion

HGA and HFOs are forms of fast neuronal population activity that have been thought to emerge in local circuits without long-range coupling beyond HGA amplitude correlations^38^ and co-occurrence of ripple oscillation bursts^3,5,6,18,19^. We report here that 100–400 Hz neocortical HFOs may be long-range phase synchronized in awake resting-state human brain activity, which indicates millisecond-accurate transmission of temporal relationships in neuronal spiking. HFO synchronization was a highly reliable physiological phenomenon and not attributable to epileptic pathophysiology or to physiological or technical artefacts. HFO synchronization exhibited a systematic connectivity pattern at the level of cortical systems defined by fMRI intrinsic connectivity^41^ and was most pronounced between the limbic and other brain systems.

Our results open a new avenue in understanding how the putative rhythmic spike synchronization in locally coherent assemblies can evoke HFO potentials in remote targets. Unlike HGA amplitude correlations or HFO burst co-occurrence, observations of HFO phase synchronization constitute direct evidence for the transmission of HFO signals per se.

Several lines of evidence suggested that HFO synchronization engaged phase coupling of rhythmic population activity rather than correlations of aperiodic, broadband HGA. The first evidence for this was the 150–210 Hz peak in the grand-average synchronization spectrum (see Fig. 2). A clustering analysis further showed that in individual subjects, peaks were discernible in the 150–210 Hz, 210–300 Hz, and 300–400 Hz frequency bands (see Fig. 3) indicating that HFO synchronization did not arise from inter-areal coupling of broadband MUA. Analysis of the similarity of phase synchronization connectomes between frequencies identified the different HFO bands as separable components in terms of their macro-scale cortical architecture (see Fig. 4). This finding was further supported by the separability of different HFO components in the micro-scale laminar profiles (see Fig. 5).

Finally, we found a visuomotor task to induce event-related HFO synchronization and desynchronization in separate frequency bands (see Fig. 8) as commonly found for task-related synchronization in lower frequencies^50^. This suggests that long-range HFO synchronization is functionally significant in visuomotor processing, which is in line with a prior study that found local synchronization of interneuronal spikes to 180–220 Hz LFP high-gamma oscillations in non-human primates to be predictive of visuomotor reaction times^12^. We found inter-areal HFO synchronization to be strongly dependent on both contacts concurrently exhibiting large-amplitude HFOs, i.e., on the internal coherence of the local assemblies^33^. HFO synchronization, both local and long-range, may thus be functionally significant in neuronal communication. We propose that the bursts of synchronized HFO oscillations observed here reflect the broadcasting and transmission of brief packets of information in large-scale neocortical networks.

Observations of stronger HFO synchronization in deep than in superficial cortical layers showed that HFOs have current sources distinct from those of low-frequency oscillations that exhibit an opposite laminar profile^28,46^ (see Fig. 5). These observations are well in line with earlier findings showing both that the ripple oscillation has maximum amplitude in layer 5 ensembles^3^ while current sources of theta and alpha oscillations are strongest in superficial cortical layers^26,51^. HFO synchronization is thus not a by-product of neuronal interactions coupling the slower oscillations, but rather a hitherto poorly understood component in the organization of large-scale brain dynamics.

Physiological HFOs and pathological pHFOs have remained non-trivial to distinguish. On one hand, pHFOs have been shown to be characteristic to epileptogenic brain tissue, to delineate the epileptogenic zone, and possibly specifically the seizure-onset zone, and to be temporally predictive of upcoming seizures^16,20–25,52^. On the other hand, a large body of studies report physiological HFOs in healthy animals^3–7^. In particular, recent studies on epileptic patients suggest that HFOs are not better biomarkers of epileptogenic tissues than epileptic spikes and that HFOs may be less specific for epileptic tissue than earlier studies have indicated and too insensitive to serve as biomarkers^53,54^. We addressed question of physiological vs. pathophysiological genesis of HFO synchronization through several lines of analyses. First, only the putatively healthy brain areas (nEZ, areas outside of the epileptogenic zone) and time-windows with no epileptic spikes were included in the primary analyses of HFO synchronization. Here, it is important to dissociate spikes (action potentials) from epileptic spikes that are massive population events with large numbers of action potentials riding a 50–100 ms wave of depolarization that may be preceded by HFOs^55^. If our HFO observations reflected pHFOs, these exclusions should greatly diminish HFO findings and, conversely, the HFO findings should be greatly more prevalent inside EZ. We did not, however, find remarkable differences in HFO synchronization in a direct comparison of nEZ and EZ areas even though low-frequency synchronization was elevated inside EZ. We also found no relationship between the rate of epileptic spikes and HFO synchronization. Finally, nEZ and EZ displayed clearly distinct phase-amplitude coupling patterns, with HFOs in nEZ dominated only by PAC with theta oscillations while HFO in EZ was coupled with both delta and theta oscillations. This is well in line with prior studies showing that pHFOs can be coupled not only to interictal spikes but also delta waves^49,56^. While in healthy freely moving rats, HFOs are exclusively phase-amplitude coupled with theta oscillations^34^. HFOs and their synchronization thus appears to be a property of healthy brain dynamics that is preserved in epileptogenic areas.

## Methods

### Data acquisition

We analyzed SEEG data from 67 subjects (age: 30.0 ± 9.4, 38 male) affected by drug resistant focal epilepsy and undergoing pre-surgical clinical assessment for the ablation of the epileptic focus. We acquired monopolar (with shared reference in the white-matter far from the putative epileptic zone) local-field potentials (LFPs) from brain tissue with platinum–iridium, multi-lead electrodes. Each penetrating shaft has 8 to 15 contacts that were 2 mm long, 0.8 mm thick and had an inter-contact border-to-border distance of 1.5 mm (DIXI medical, Besancon, France). The anatomical positions and amounts of electrodes varied according to surgical requirements^57^. On average, each subject had 17 ± 3 (mean ± standard deviation) shafts (range 9–23) with a total of 153 ± 20 electrode contacts (range 122–184, left hemisphere: 66 ± 54, right hemisphere: 47 ± 55 contacts, gray-matter contacts: 113 ± 16.2). We acquired an average of 10 min of uninterrupted spontaneous activity with eyes closed in these patients with a 192-channel SEEG amplifier system (NIHON-KOHDEN NEUROFAX-1100) at a sampling rate of 1 kHz. Before electrode implantation, the subjects gave written informed consent for participation in research studies and for publication of results pertaining to their data. This study was approved by the ethical committee (ID 939) of the Niguarda Ca’ Granda Hospital, Milan, and was performed according to the Declaration of Helsinki.

### Signal pre-processing

We excluded electrode contacts (1.3 ± 1.2, range 0–10, contacts) that demonstrated non-physiological activity from analyses. We employed a referencing scheme for SEEG data where electrodes in gray matter were referenced to the closest contacts in white-matter (cWM)^28^. This referencing scheme both yields signals with a consistent polarity and limits the mixing of signals from active sources to provides more accurate phase estimates.

Prior to the main analysis, SEEG time series were low-pass filtered with FIR filter with cutoff at 440 Hz and stop-band at 500 Hz (60 Hz transition band, −6dB suppression at 475 dB, maximal ripples in pass-band 2%). Fifty hertz line-noise and its harmonics were excluded with a band-stop FIR filter with 53 dB suppression and 1 Hz band-stop widths. The LP filtered data were then separated into narrow frequency bands with 50 Morlet wavelets of (width *m* = 7.5) and frequency ranging from 2 to 450 Hz.

Epileptic events such as interictal spikes are characterized by high-amplitude fast temporal dynamics and widespread spatial diffusion. Owing to possible filtering artefacts around epileptic spikes and the resultant increase in synchrony, we discarded periods of 500 ms containing Interictal Epileptic Events (IIE). We defined such periods as the temporal windows where at least 10% of cortical contacts demonstrated abnormal concurrent sharp peaks (amplitude envelope peaks exceeding five times the standard deviation of the contact mean amplitude) in more than half of the 50 frequency bands.

### Defining the epileptic zones from seizure activities

The epileptogenic and seizure propagation zone were identified by clinical experts in a visual analysis of the SEEG traces^57,58^. Epileptogenic areas are the hypothetical brain areas that are necessary and sufficient for the origin and early organization of the epileptic activities^59^, from where contacts recording often show low-voltage fast discharge or spike and wave events at seizure onset. Seizure propagation areas are recruited during the seizure evolution, but they do not generate seizures^60,61^, from where contact recording show delayed, rhythmic modifications after seizure initiation in the epileptogenic areas. In this study, we combined epileptogenic and propagation areas as the epileptogenic zone (EZ) to distinguish from the rest of brain areas that are exhibit putatively healthy active (non-epileptogenic zone, nEZ).

### Functional connectivity estimates

We estimated inter-areal phase interactions at individual subject level using the phase-locking value (PLV). Defining *x*′(*t*) as the complex wavelet coefficients for a given frequency of the signal *x*(*t*), complex PLV (cPLV) is computed as

1 $${\mathrm{cPLV}} = \frac{1}{T}\sum_{t = 1}^T {\frac{{x{^\prime}(t)}}{{\left| {x{^\prime}(t)} \right|}}\frac{{y{^\prime}^ \ast (t)}}{{\left| {y{^\prime}(t)} \right|}}},$$

where *T* is the sample number of the entire signal (i.e., ~10 min), and * is complex conjugate. We computed cPLV for the entire recording excluding the 500 ms time windows showing epileptic or artefactual spikes (see above). The PLV is the absolute value of cPLV (PLV = |cPLV|), and it is a scalar measure bounded between 0 and 1 with indicating complete phase synchronization.

Additionally, we used absolute imaginary part of cPLV (iPLV = |Im(cPLV)|), a metric insensitive to zero-lag interactions caused by volume conduction^62,63^, to quantify phase lagged phase synchronization as a control analysis that is insensitive to linear mixing caused by volume conduction. For both PLV and iPLV connectivity, we denote the fraction of significant connections (*K*) as the number of SEEG contact pairs exhibiting significant phase synchronization divided by the total number of contact pairs. All such pairs of SEEG contacts that shared the white-matter reference contact were excluded from all analyses.

### Cluster analysis of phase synchronization profiles

The sparsity of the mesoscopic field-potential signals picked up by the SEEG electrode contacts and the anatomical heterogeneity of SEEG implantations across subjects make it possible that group averaging conceals robust individual physiological patterns or is influenced by outlier individuals. To assess individual HFO synchronization patterns and the presence of outliers, we divided the subjects into clusters by using hierarchical clustering with Ward’s method for merging the branches^64^. Following this method, a new cluster is computed as an argmin of the error sum of squares after merging given pair of clusters:

2 $${\mathrm{{\Delta} }}\left( {{\mathrm{A}},{\mathrm{B}}} \right) = \mathop {\sum }\limits_{i \in {\mathrm{A}}\mathop { \cup }\nolimits^ {\mathrm{B}}} \left| {\left| {\overrightarrow {{\mathbf{x}}_i} - \overrightarrow {{\mathbf{m}}_{{\mathrm{A}}\mathop { \cup }\nolimits^ {\mathrm{B}}}} } \right|} \right|^2 - \mathop {\sum }\limits_{i \in {\mathrm{A}}} \left| {\left| {\overrightarrow {{\mathbf{x}}_i} - \overrightarrow {{\mathbf{m}}_{\mathrm{A}}} } \right|} \right|^2 - \mathop {\sum }\limits_{i \in {\mathrm{B}}} \left| {\left| {\overrightarrow {{\mathbf{x}}_i} - \overrightarrow {{\mathbf{m}}_{\mathrm{B}}} } \right|} \right|^2 = \frac{{n_{\mathrm{A}} \ast n_{\mathrm{B}}}}{{n_{\mathrm{A}} + n_{\mathrm{B}}}} \ast \left| {\left| {\overrightarrow {{\mathbf{m}}_{\mathrm{A}}} - \overrightarrow {{\mathbf{m}}_{\mathrm{B}}} } \right|} \right|^2,$$

where $\overrightarrow {{\mathbf{m}}_{{j}}}$ is the center of cluster *j*, and *n*_*j*_ is the number of points in it and Δ(A, B) is called the merging cost of combining the clusters A and B.

Elbow method was used to determine the optimal number of clusters^65^. We obtained a series of cost of merging for each number of clusters and computed the optimal group count as the point with the largest drop of cost in respect to previous gain.

### Statistical hypothesis tests

We estimated the null hypothesis distributions of interaction metrics with surrogates that preserve the temporal autocorrelation structure of the original signals while abolishing correlations between two contacts. For each contact-pair, we divided each narrow-band time series into two blocks with a random time point *k* so that **x**′_1_(*t*) = **x**′(1…*k*) and **x**′_2_(*t*) = **x**′(*k*…*T*), and constructed the surrogate as **x**′_surr_(*t*) = [**x**′_2_, **x**′_1_]. We computed surrogate cPLV (PLV and iPLV) across all channel pairs and their mean and standard deviation were later used in hypothesis testing.

To test significance of PLV differences in distance bins for a single frequency we computed Spearman correlation between distance bin label and actual PLV over bootstrapped data (number of bootstraps = 1000) and compared the observed negative correlations with the 5th percentile of minimum correlation of shuffled data (number of shuffles = 1000).

Similar method was used to test PLV difference for nEZ–nEZ and EZ–EZ contacts in distance bins: we computed the difference between contacts recorded from EZ and nEZ gray matter loci for bootstrapped data (number of bootstrap rounds = 1000) and compared it with 95th percentile of maximum difference for shuffled data (number of shuffles = 1000).

### Post-processing of the phase synchronization analyses

To assess how interaction strength varies as a function of spatial distance between recording sites, we divided the inter-contact distances into four ranges: *very short* below 32 mm; *short*: between 32 and 45 mm; *medium*: between 45 and 60 mm; *long*: >60 mm. Each range encompassed the same number of subject inter-contact edges, i.e., 368,043/4 = 92,011. The confidence intervals for PLV and iPLV, were expressed relative to the surrogate means (SM) for PLV (3.42*SM corresponding to *p* < 0.001, Rayleigh distribution), and the surrogate standard deviations (SD) for iPLV (3.58*SD corresponding to *p* < 0.001, normal distribution).

To compare signals from superficial and deep layers in gray matter (Fig. 4 and Supplementary Fig. 3), we divided contacts into superficial and deep groups based on their Gray Matter Proximity Index (GMPI)^28^ that is defined as the relative distance between the contact location and the nearest white-gray border surface, normalized by the gray matter thickness at that location:

3 $${\mathrm{GMPI}} =[(C - W) \cdot (P - W)]/\left| {P - W} \right|,$$

where *P*(x, y, z), *W*(x, y, z), and *C*(x, y, z) are the vertices on the pial, white-matter surface, and contact coordinates in 3D individually reconstructed brain from MRI scan, respectively. Values 0 < GMPI < 1 indicate that the contact midpoint is located in gray matter, whereas a negative GMPI indicates that the contact midpoint is in the white-matter.

We set the criteria −0.3 < GMPI < 0 and 0.5 < GMPI < 1.2 to classify deep and superficial layer contacts, respectively^28^. Next, PLV and iPLV estimates were averaged across subjects between deep-deep (D–D) and superficial–superficial (S–S) contact-pairs. We tested for between-groups difference with a paired permutation test (100 random samples created by shuffling S–S and D–D labels within subjects; threshold for significance corrected for multiple comparisons with Benjamini–Hochberg method at *α* < 0.05).

### Anatomical localization of the SEEG electrode contacts

To assess the neuroanatomical structure of inter-areal and inter-system HFO synchrony at group level, the electrode contact locations were expressed in terms of a standard brain atlas. We used the Schaefer parcellation at 100-parcel resolution^42^ where each parcel is assignable to one of seven Yeo functional systems^41^. The parcellations were created using individual pre-surgical T1 MRI 3D-FFE scans and the Freesurfer^66^ software.

### Phase-amplitude coupling of slow and fast rhythms

Two neuronal oscillations are cross-frequency phase-amplitude coupled (PAC) if the phase of the slower oscillation is correlated with the amplitude of the faster^67,68^. We estimated PAC with the phase-locking value (PLV) as:

4 $${\mathrm{PLV}} = \left| {\frac{1}{N}\sum_{n = 1}^N {{\mathrm{e}}^{{\mathrm{i}}\left( {\theta _{{\mathrm{amp}}}(n) - \theta _{{\mathrm{phase}}}(n)} \right)}} } \right|,$$

where we first filtered the amplitude envelope of the fast rhythm with the filter used for the slow rhythm and acquired its phase time series as *θ*_amp_(*n*) and then assessed its correlation with is the phase time series of the slow rhythm, *θ*_phase_(*n*). The significance of PAC PLV value was determined in the same manner in individual subjects as for PLV used for measuring phase synchrony above.

### Phase synchronization dependence on amplitude correlations

To assess the relationship of HFO phase synchrony and HFO amplitude values, we used the instantaneous amplitude and phase values of the Morlet-filtered time series. The amplitude values were divided into quintiles and for each frequency and pair of SEEG electrode contacts exhibiting significant HFO synchronization, we compiled an amplitude-amplitude matrix containing the instantaneous contact-pair phase differences for time-samples corresponding to the associated combination of amplitudes in quintile bins. The number of samples in each bin yielded a measure of amplitude correlations because at the null hypothesis of no correlations, the amplitude-amplitude matrix is uniform because each quintile contained the same number of samples (see Fig. 6a). To quantify the amplitude correlation, we evaluated the difference of the observed distribution from a uniform distribution for each amplitude-amplitude bin and used the mean difference (in % change) across bins (see Fig. 6b). To quantify whether HFO phase synchrony was correlated with the moment-to-moment amplitude relationships, we quantified PLV across the phase differences in each amplitude-amplitude bin (see Fig. 6c) after equalizing the numbers of samples across bins by randomly discarding samples exceeding the smallest number of samples. We further quantified the dependence of HFO synchrony on the joint amplitudes by measuring the mean difference (in %) of the observed PLV distribution from a uniform PLV distribution (mean of the observed PLV values) expected at the null hypothesis of no relationship between HFO and amplitude (see Fig. 6d).

### Time-resolved phase synchronization

To test whether HFO phase synchrony was dynamically recruited in task-relevant neuronal circuitry during a visuomotor task. We inspected time-resolved phase synchronization^69^ with an addition cohort of *N* = 11 patients who performed a visuomotor Go/NoGo task^70^. The subjects were instructed to respond with a key press as quickly as possible to visual Go (750 events) stimuli and withholding their responses the NoGo (250 events) stimuli that were presented randomly with a fixed 1 s inter-stimulus interval (for details, see ref. 75). One subject was excluded due to excessive artefacts and one for making no responses during the experiment. In the remaining nine subjects we rejected an average of 33.9 ± 26.6 trials containing technical artefacts or epileptic spikes based on visual inspection of raw traces.

Go/NoGo SEEG data were preprocessed identically to the main cohort resting-state data, using cWM-referencing and the same 18 HFO-range Morlet filters to quantify time-resolved phase synchronization. We assessed event-related neuronal responses for Go trials with responses (724.9 ± 20.6, range = 682–750) and NoGo trials with withheld responses (241.2 ± 6.2, range = 230–250). The recording was partitioned into 1500 ms epochs, i.e., 500 ms prior to, and 1000 ms after cue onset. To avoid filtering artefacts, we rejected the first and the last 100 ms of samples for each trial (longest Morlet wavelet was 91 samples in length). Narrow-band HFO amplitude envelopes were average across trials and then subtracted the mean baseline amplitude (400–10 ms prior to cue). We then normalized (*z*-score) these baseline-corrected responses, averaged across 18 frequencies and defined task-relevance for each contact based on the global high-gamma amplitude response averaged during 100–400 ms after cue onsets (one exemplary subject see Fig. 8a).

For time-resolved HFO synchrony analysis, we divided each trials into 130 non-overlapping windows of 10 ms duration, i.e., each epoch contained samples −400 to 900 ms around cue. We computed cPLV (Eq. (1)) by averaging phase differences within each time window across trials. We next averaged cPLV of every 20 consecutive windows with overlap of 10 windows to obtain cPLV in long windows of 200 ms duration. Time-resolved cPLV surrogates between two contacts were constructed by randomly shuffling trials from one contact while keeping the original trial order from other contact.

To robustly assess the functional relevance of time-resolved HFO synchrony during task, we performed a cluster-based analysis on the time-frequency representation (TFR) of the baseline-corrected PLV (ΔPLV). We first averaged the frequency representation of the baseline-corrected and time-resolved PLV between the 5% most responsive contacts (Fig. 8b). We next binarized these channel-pair averaged TFR PLV (Fig. 8c) by applying 100 PLV thresholds ranging from 0.0001 to 0.008, i.e., up to no PLV values above threshold. Finally, for each possible threshold, we identified positive (PLV > 0) and negative (PLV < 0) clusters in TFR PLV with connected components, i.e., portions of TFR binary mask sharing common neighbors (Fig. 8d). For each cluster we computed its area as the number of elements in it and took as descriptive statistics the sum of areas for the three largest positive and negative clusters in each subject. We summed the areas of the three largest positive and negative clusters for each threshold and defined the effect size as the area-under-curve between the real and surrogate cluster sizes as a function of thresholds for both positive and negative clusters (Fig. 8e). Finally, effect sizes are averaged across subjects. We computed the group average effect size for 16 possible relative number of contacts (step equals to 1 percent from 2 to 10, and 2% from 10 to 25) and selected the percentage that showed a peak in positive responses clusters (Fig. 8f). Finally, to test the hypothesis that time-resolved PLV increase was prominent only among the task-relevant contacts, we estimated effect size in groups of 5% contacts as function of their position in sorted average envelopes profiles (see Fig. 8a for an example of the result for one subject). Specifically, we sorted contacts based on their amplitude response, and defined their position as the fractional index in the sorted array. To assess significance, we compared effect size for subsets of 5% channels along their positional index in high-gamma bands with equal sampled subsets of randomly selected channels along the same axis (number of shuffles is equal to 100). Finally, we quantified the fraction of subjects (*K*) showing effect size above confidence interval at 95%.

### Reporting summary

Further information on research design is available in the Nature Research Reporting Summary linked to this article.

## Supplementary information

Supplementary Information

Reporting Summary

## Data Availability

Raw data and patient details cannot be shared due to Italian governing laws and Ethical Committee restrictions. Intermediate as well as final processed data that support the findings of this study are available from the corresponding authors upon reasonable request.

## Code availability

All code used in this work to produce the connectome matrices, to create figures, and to run the statistical analyses can be found at https://github.com/palvalab/seeg_hfo_synchronization/.
