# Supplementary material for: Long-range phase synchronization of high-frequency oscillations in human cortex
Source: Nat Commun. 2020 Oct 23;11:5363. doi: 10.1038/s41467-020-18975-8 (PMC7584610; doi:10.1038/s41467-020-18975-8)
Supplement: Supplementary file 1 — Supplementary Information [file 41467_2020_18975_MOESM1_ESM.pdf]

Supplementary Information for Submitted Manuscript

Long-range phase synchronization of high-frequency oscillations in human cortex

Arnulfo G, Wang SH, Myrov V, Toselli B, Hirvonen J, Fato MM, Nobili L, Cardinale F, Rubino A, Zhigalov A, Palva S, Palva JM

Supplementary Methods

Using PLV and iPLV for measuring phase synchrony

The phase-locking value (PLV) is a metric for measuring phase synchrony between pairs of physiological signals. The PLV estimates derived from on-scalp macroscopic measurement of neuronal activity, such as MEG and EEG, are inflated by linear mixing caused by volume conduction or field spread [1–3]. The imaginary part of the complex-valued cPLV (iPLV) is insensitive to zero-phase-lag interactions and therefore is not inflated by linear mixing. The iPLV is, however, also insensitive to true near-zero-lag neuronal phase coupling. Here, we used PLV as the main metric and iPLV only for validation because the SEEG signals are highly local, especially in the HFO frequencies, and the effects of volume conduction are limited by referencing.

The choice of referencing schemes for SEEG data

We used here a referencing scheme that exploits the nearest white matter (cWM) SEEG contacts as silent references for the grey-matter contacts [4]. This referencing scheme effectively attenuates volume conduction and provides more accurate assessment of cortical phase dynamics than using bipolar referencing because it does not mix active neuronal sources in grey matter.

In bipolar referencing, each SEEG contact is referenced to its immediate neighboring contact on the same electrode shaft. With a center-to-center separation of 3 mm between two neighboring contacts, bipolar signals therefore reflect highly local neuronal activities [4,5]. In this study, the contact edge-to-edge distance was 1.5 mm and the contact dimensions was 2 mm, which was comparable to the average cortical thickness of 2.5 mm in adult human brains. Thus, bipolar referencing could mix neuronal sources in superficial and deep cortical layers [4]. Moreover, when two neighboring contacts happened to be implanted in two functionally distinct regions, bipolar referencing could distort signals thus misinform later analyses and interpretations. Although we considered bipolar referencing less optimal than the cWM referencing, for validation, we conducted the same set of analyses with both referencing schemes, *e.g.*, phase synchrony spectrum (Fig. 2) and layer profiles (Fig. 5).

Morphing individual inter-contact PLV into group-level connectivity

To study the group-level neuroanatomy of HFO synchrony (Fig 5), we assigned each SEEG electrode contact to a Schaefer parcel (see Methods) and then morphed the inter-contact PLV into a group-level connectivity map. For this analysis, we used cWM referenced data from cluster 1 and 2 subjects (N=49) who showed HFO peaks in PLV/iPLV spectra (Fig 3). We used only contact pairs that were recorded from non-epileptic, neocortical grey matter loci. The morphing was done by first averaging within subjects the contact-pair PLVs falling into same parcel pairs, and then averaging across subjects to obtain the group averages of the inter-parcel PLVs. By doing so, we thus collapsed

sparsely sampled, patient-specific inter-contact PLV into well-sampled, population-level inter-parcel (inter-areal) phase synchrony networks (sampling stats see Fig. 1). In addition, we excluded a small number (8% of total inter-contact PLV) of individual putative outlier inter-contact observations with PLV > 0.25.

Handling missing data for modularity estimates

Due to the low inter-hemispheric coverage, we limited our investigation to intra-hemispheric modular structures. Within hemispheres, 10–20% of possible parcel pairs remained unsampled (Fig. 1c, Suppl. Fig 5). To circumvent the problem of missing connectome values, we detected modules in the synchrony profile similarity matrix (examples see Fig 4) that was a second-order functional connectivity matrix derived from the inter-parcel PLV matrices. Each element in the synchrony similarity matrix was obtained by taking the connectivity profiles, *i.e.*, row vectors from the inter-parcel PLV matrix, of each pair of Schaefer parcels and computing the Spearman correlation coefficient between these rows considering only the values that were sampled in both rows. This similarity matrix thus denotes how similar two parcels were in terms of their connectivity with the rest of the network. This measure is robust against noise and missing values.

Detecting the community structures in the HFO synch similarity matrices

To investigate whether the HFO synchrony networks can be reliably subdivided into well-separated functional modules of nodes (communities) across frequencies, we applied the Leiden algorithm [6] that uses a resolution parameter γ to weigh the importance of the null model, *i.e.*, random network with no modular structure, against which the original network was compared. When detecting modules in a connectivity graph, the algorithm maximizes the modularity value:

$$Q(\gamma) = \frac{1}{2m}\sum_{i,j}\left[\mathbf{A}_{i,j} - \gamma p_{i,j}\right]\delta(\sigma_i, \sigma_j) \qquad (1)$$

where $Q(\gamma)$ is the modularity at resolution γ; m is the total strength of the network, $A_{i,j}$ is the network value in row i and column j, while $p_{i,j}$ are the network values in the null model, i.e. expected by chance for row i and column j. Here, $p_{i,j} = \frac{k_i k_j}{2m}$, where k_i and k_j are the total strength of network values in row i and column j respectively; $\delta(\sigma_i, \sigma_j)$ is equal to 1 when node i and node j belong to same community, and 0 otherwise.

Thus, $Q(\gamma)$ quantifies the quality of the network partition: a good partition should maximize the connections between nodes in the same module, while minimizing the connections between nodes in different modules. Values of γ less than 1 tend to favor a small number of large communities, since the null model was down-weighed; on the contrary, values of γ larger than 1 tend to produce a high number of small communities, since the importance of the null model was up-weighted. We detected modules for each of the synchrony similarity matrix using γ ranging from 1 to 1.5 with steps of 0.05. We then assessed the range of resolution parameter values at which modules could be reliably identified.

As a preliminary analysis, we estimated modularity (Supplementary Equation 1) as we tuned the resolution parameter γ when detecting modules in narrow-band PLV connectivity maps (Supplementary Figure 6), *i.e.*, resolution varied from coarse to fine as γ increased, which resulted in the Leiden algorithm partitioning a connectivity map into increasing numbers of modules. Because we used weighted graphs for module detection (Supplementary Equation 1) and the PLV estimates varied across frequency bands (Fig 2), the estimated modularity needed to be normalized. Thus, for each narrow-band frequency and γ value, the observed modularity was normalized by computing a z-score using a null distribution of modularity values, *i.e.*, obtained by applying the Leiden algorithm with the same γ value to 100 null hypothesis (H_0) synchrony similarity matrices. These H_0 similarity

matrices were obtained by randomly rewiring the network with the edges of the original synchrony similarity matrix while keeping the degree and edge-weight distribution intact. In both hemispheres, across narrow-band HFO frequencies and throughout tested resolutions, the modularity of the observed PLV connectivity maps was significantly larger than that of H_0 graphs and most pronounced in 150–270 Hz range, which we identified as the band of interest (permutation test, $p<0.05$, N=100, one-tailed, Supplementary Figure 5d).

Evaluating module assignment stability across brain regions and resolutions

After module detection in HFO connectivity maps, we next evaluated the 'stability' of the module assignment to each Schaefer parcel (Fig. 4). The goal was to assess how consistently each parcel was assigned to a specific module at a specific resolution γ. For any narrow-band (Supplementary Figure 6) or band-collapsed (Fig. 4) HFO connectivity map to be tested at a specific resolution γ, the estimate of stability was conducted as follows [7]:

1. We first generated 100 bootstrap copies of the original connectivity map by bootstrapping subjects with replacements.
2. We next computed the synchrony similarity matrix for each of the bootstrapped PLV matrices and then detected modules at a specific resolution (Methods). Thereby, for each parcel P_i and resolution parameter γ, we obtained a module co-assignment vector of 50 zeros or ones, where one indicates whether another region was assigned in the same module as P_i.
3. This co-assignment vector of P_i from the bootstrap instance was compared to co-assignment vector of P_i from original connectivity map. In particular, we estimated the sum of the number of regions that were correctly located in the same module and the number that was correctly located in other modules (compared to the original modular structure), as a function of the total number of regions (N=49, *i.e.*, excluding self-referencing). This was done for each of the 100 bootstrapped synchrony similarity matrices and the stability for parcel P_i is a real number between 0 and 1, quantifying the similarity of the modular structure between the original and bootstrapped networks. A value close to 1 indicates high stability of the module assignment to P_i the original connectivity map.
4. Finally, the stability estimate of a parcel P_i was considered as significant if it was greater than the 95%-tile of the null-hypothesis distribution of stability estimates derived from 100 instances of randomly shuffled original assignment vector of P_i (corrected for multiple-comparison for 50 brain regions for two hemispheres independently).

Ruling out technical confounders from the amplifier

At the beginning of 2017, the Niguarda Hospital renewed the amplifiers and acquisition system to a more recent version from Nihon-Kohden (Neurofax-1200A). An additional cohort (N=19) was acquired with the new amplifier, while the main cohort (N=67) was acquired with the prior one. To quantify the amplifier noise level and profile and as an additional control experiment for putative artefactual origins of HFO synchrony, we obtained 10 minutes of recording from two electrodes (each with 18 contacts) immersed in a saline solution. We computed the average power spectral densities, by means of the modified periodogram using Welch's method of the bipolar referenced saline solution recordings and resting-state recording of the new-amplifier-cohort (Supp. Fig. 3 e-g). Finally, we quantified the phase synchronization spectrum for both real and saline-solution data (see Fig. 2d).

Simulation based assessments of line-noise effects in phase reconstruction accuracy

Neuronal activity in HFO range has low amplitude, and therefore, leakage of line-noise (50 Hz in EU) and its harmonics into the recorded signal at HFO frequencies could potentially impact our results. In general, band-suppression filters have a narrow stopband to limit information loss and a high attenuation coefficient to maximize suppression effects. These parameters in discrete-time system design frequently result in a high order filter with steep transition bands. The latter could

introduce spurious activity and reduce temporal accuracy. Here, we used Finite Impulse Response (FIR) filters that have a linear phase response and thus do not alter phase of the reconstructed signals and are stable linear systems. FIR filters are generally stable by definition, but their design usually results in a higher-order system compared to Infinite Impulse Response (IIR) filters. Hence, we directly tested the effects of line-noise to the reconstructed phase time series with our filter design. Low accuracy in phase reconstruction after line-noise filtering could thus potentially bias results at high-gamma range. Therefore, we first simulated a set of oscillatory signals identical to that in the previous test for the Nyquist limit, and then we added a 1/f noise for each of the simulated center frequencies (Supplementary Figure 4 a, b). Finally, we simulated very narrow band 50, 100, 150, …, 300, and 350 Hz oscillations to mimic the line-noise signal and harmonics (Supplementary Figure 4c). With these data we tested phase reconstruction accuracy by quantifying the PLV between original oscillations and filter-reconstructed and line-noise-suppressed signals.

Simulation based assessments of Nyquist limit in phase reconstruction accuracy

The Nyquist–Shannon theorem defines a sampling rate that allows the information in a continuous signal with finite bandwidth to be captured by a discrete samples time series. When the bandwidth is close to half of the sampling frequency, *i.e.*, the Nyquist limit, aliasing arises. In discrete-time signal processing, aliasing is an effect that causes subsequent frequency harmonics to overlap with main frequency content. This overlap generates artefacts in both time and frequency representation of the signal that render different signals indistinguishable when inappropriately sampled. Given the novelty of our results and that the upper boundary of the high-gamma band resides close to the Nyquist limit (in our data 500 Hz), we assessed whether aliasing could possibly have inflated our current observations.

To assess the phase reconstruction accuracy in wavelet frequencies near the Nyquist limit, we simulated narrow-band time series with temporal dynamics resembling those of biological signals. First, we generated four independent Gaussian white noise (25 seconds with a sampling frequency of 5kHz); we next filtered each time series with Morlet wavelets (7.5 cycles wide) centered at 100Hz, 200Hz, 300Hz, and 400Hz, respectively; the filtered narrow-band time series were next linearly combined and then embedded in an independent uniform background noise (Supplementary Figure 5a). For each simulated center frequency, we extracted the phase time-series of both the simulated (original) and filter-reconstructed signals (Supplementary Figure 5b) and then computed the phase locking value (PLV) between them. Finally, to show that phase reconstruction accuracy was independent from sampling frequency, we also compared full-resolution (5kHz) and down-sampled (1kHz) versions of the simulated oscillations.

Supplementary Notes

SEEG spatial sampling statistic

SEEG invasive recordings yields sparse sampling of the brain volume and implantation strategies are driven solely by clinical requirements on individual basis. In our main cohort (N=67), the majority of the patients had unilateral implantation of the SEEG electrodes, and 11 subjects had bilateral implantation. Although the number of non-epileptic (nEZ) cortical contacts per patient (b) and distinct subject sampled in each cortical parcel (c) varied, contact number and fraction of subject per parcel were correlated (r = 0.9), thus ruling out the possibility that a subset of subjects is biasing the parcel-to-parcel connectivity estimates. On the systems-level the right hemisphere was sampled more extensively than the left hemisphere (d), but the majority of the left hemisphere functional systems were sampled with more than 20 distinct subjects. Therefore, the connectivity was well sampled on the systems-level as well.

For the large-scale connectivity analyses (Fig 2), we only included contacts located in healthy (nEZ) regions. Due to the large cohort size, we also obtained adequate numbers of contacts in epileptogenic zone (EZ) (Supplementary Figure 1), which enabled comparisons of cross-frequency coupling and within-frequency synchrony between nEZ and EZ (Fig. 7).

Controlling for possible contribution from artificial causes to HFO synchrony

HFO synchrony was not caused by a specific referencing scheme or volume-conduction

Overall, analysis of HFO synchrony in both cWM and bipolar referenced data using PLV and iPLV yielded converging results (Fig 2 and Supplementary Figure 1 g–h). The mean PLV, iPLV, and corresponding connection density (K) estimated from bipolar referenced data showed no visible spectral differences from that of cWM referenced data, thus ruling out the possibility that HFO synchrony was due to a specific referencing scheme. Importantly, the iPLV values were highly significant across all high-gamma frequencies, hence a vast majority of the HFO synchrony had non-zero phase lag.

In summary, the additional analyses conducted using bipolar referencing and iPLV rule out the possibility that the HFO synchrony could be attributable to extra-cranial sources of physiological artefacts, such as eye or scalp muscle activities, or external device related noise patterns. Such sources of non-neuronal origin could not produce electric field gradients steep enough to be observable in bipolar recordings while maintaining systematic long-range non-zero-lag phase relationships between widely separate and highly local cortical SEEG signals.

Split-cohort reliability of HFO synchrony

We tested the split-cohort reliability of PLV strength and connection density (K) of the HFO synchrony on contact-level and systems-level. We randomly split the subjects into two cohorts (34, 33 subjects respectively) so that the number of contacts per region in the two cohorts was maximized. Both the large-scale mean PLV, between-system PLV, and K were reliable, *i.e.*, high correlations of the split cohorts to original data (Supplementary Figure 2 a-d, see Fig. 2 a, b) as was the pattern of functional connectivity between cortical layers (Supplementary Figure 2 e-f). It appears unlikely that the across-subject reproducibility of large-scale cortical architecture could arise from technical artefacts or extra-cranial sources in a cohort where each subject has a unique pattern of SEEG shaft implantation.

Replicating HFO synchrony with an independent dataset

Recently, Miller K. shared a large intracranial-EEG database that includes electrocorticography (ECoG) time-series sampled at 1kHz and 10kHz during a series of different experiments [8,9]. We analyzed those data with our pipeline in order to see whether we could replicate our finding with data collected with different hardware setups. Miller's database included 16 different recording sessions and 34 different patients for a total of 204 distinct datasets. We rejected by visual inspection 3 subjects and 3 conditions due to poor SNR in the HFO frequency range or large contamination of multiple artefacts. Moreover, we filtered the data with a 300Hz low-pass filter (instead of the 440Hz filter used in main analyses) to further attenuate the possible effects of high-frequency artefacts present in several channels. We then re-referenced each channel to its closest neighbor to construct a bipolar scheme that is equivalent to what we used in our SEEG data. After filtering with identical filter banks of 50 Morlets and line-noise-suppression filters that were used in our analysis, we computed iPLV and fraction of significant K(iPLV) for all recording conditions and all subjects. We concluded that, using Miller's ECoG data, we were able to replicate robust HFO phase synchrony seen in our SEEG data (Supplementary Figure 2 j-m). The observation of HFO synchrony in Miller's ECoG data offers

an important support to the generalizability of HFO phase synchrony being a global property of the human brain.

HFO synchronization is not attributable to artefactual sources

The connectivity and community structures of HFO synchronization as well as its laminar organization strongly suggest that it may not be attributable to physiological or technical artefacts such as signals from muscles or extra-cranial sources. Nonetheless, we corroborated this notion with a number of controls. First, in addition to white-matter referencing, HFO synchronization was observable with bipolar referencing (Fig. 2c, Supplementary Figure 1 g-h), indicating that its current sources are millimeter-scale local in cortical tissue and very unlikely to originate from extracranial or muscular sources during inter-ictal events[10]. Second, HFO synchronization was also observable with linear-mixing insensitive interactions metric and hence not attributable to volume conduction [11] (Fig. 2b, Fig. 3d–f). Third, HFO synchronization was comparable among electrodes along single electrode shafts and between electrodes in different shafts (Supplementary Figure 3 a), which excludes the contributions of implantation-related lesions along the shafts as well as voltage diffusion attributable to excessive perfusion of cerebrospinal fluid (CSF) in the cortical lesion following shafts implant. Fourth, similar observations were made with two different SEEG data-acquisition systems and the recordings with the same electrodes in saline solution did not show any indication of artificial HFO synchronization. HFO coupling thus does not arise from the signal amplifier or data-acquisition electronics.

We conducted further control analyses to rule out that artefacts within single electrode shaft had no effect on observed HFO synchrony. Such artefacts could be caused by CSF entering cerebral tissue along the insertion trajectory. The CSF is a pervasive medium that is characterized by an electrical conductivity that is estimated on average as 1.79 S/m in the human brain, far greater than the conductivities of grey and white matter, 0.47 S/m and 0.22 S/m, respectively[12]. SEEG electrodes were implanted by percutaneous twist-drilling of the skull and inserting a guiding stylet that dissects through the cerebral tissue all along the planned trajectory. The stylet is immediately replaced with a multi-lead electrode shaft uniquely identified with a capital letter and a running index starting from the tip (*e.g.*, A1…A#). Robot-assisted surgery and clinical experience have made the implantation technique of SEEG electrodes performed at Niguarda Hospital safe, with a complication rate of only 1.8% [13]. The amount of CSF leakage is very small, thus neglectable [14,15]. Nonetheless, stylets might ease the flow of CSF through the insertion trajectory and resulting in bridging artefacts, *i.e.* promoting field spread between neighboring contacts along the same shafts. If that would be the case, we would observe increased levels of high-gamma synchronization between electrode contacts within the same shaft compared to those between shafts. Therefore, we compared within- and between- electrode shafts connectivity. We observed that HFO synchrony profiles between- and within-shafts largely reproduce main effect *i.e.* PLV spectral profiles show increase in HG range.

The with- and between-shaft contacts showed no difference in HFO synchrony in very-short, short, and medium distance (Supplementary Figure 3a). In slower frequencies from 2 to 100Hz, within-shaft phase synchrony was increased in very-short, short and medium ranges. Very-short range connectivity is known to be affected by volume conduction and suggesting that CSF could have played a role in the increased within-shaft PLV. Furthermore, long-range phase synchronizations were more pronounced between-shafts than within-shafts suggesting that the long-range HFO synchrony could not be caused by within-electrode artefacts. Moreover, we found that the iPLV spectral profile was similar to that of PLV, which further rules out the possibility that HFO synchrony could be caused by CSF leakage.

Taken together these results prove that the reported observations of HFO synchronizations cannot be explained by within-shaft artefacts such as field spread promoted by CSF leakage along the electrode insertion trajectory.

No evidence of muscular activity contributions to HFO synchrony

We hypothesized that, if muscular activity inflated the HFO phase synchrony, the contacts closer to the skull would have picked a larger fraction of muscular activity compared to contacts recording far from it [16]. Hence, we analyzed separately contacts recording far-from-skull (contact numbers from 1 to 6) from those that are near-skull (contact numbers > 10). We observed that closer-to-skull contacts showed no significant increase in phase synchrony compared to far-from-skull contacts (Supplementary Figure 3b) suggesting absence of confounds from muscular artefacts.

Inter-ictal events were not correlated with HFO synchrony

Theoretically, transient spike-like neuronal events could be picked up by filtering in the HFO frequency band and cause artefactual HFO synchronization. We aimed to discard all time-windows containing epileptic spikes (referred to as cleaned-data, see main text and Methods) but to assess whether residual subthreshold spikes could confound the results, we assessed the correlation between spike rate and HFO synchrony. We found that the number of spikes detected was not correlated with the mean PLV across frequencies except for 2.5 Hz (Supplementary Figure 3d). The lack of correlation rules out the possibility that HFO synchronization was confounded by the inter-ictal events.

No synchrony between contacts recorded within saline solution

To investigate whether HFO could artefactually arise from the acquisition system, we also estimated the phase-synchronization for SEEG contacts recorded in saline solution and hence with the absence of any brain activity. We did not observe phase synchronization between SEEG contacts recorded within saline solution in any of the frequencies (Fig. 2d). Furthermore, the strength of between-contact phase synchronization obtained from saline solution was close to the surrogate data computed for real patient data but smaller than the strength of HFO synchronization (see red dashed line in Fig. 2d). The observed HFO synchrony cannot thus be due to artificial sources from the acquisition system. (Supplementary Figure 3 e-g)

Changes in Signal-to-noise ratio did not play a role in HFO phase synchronization

Changes in signal-to-noise ratio could play a role in the detectability of neuronal activity and therefore also in phase synchronization estimates because the accuracy of phase estimation is dependent on the signal-to-noise ratio (SNR)[17]. Hence, we asked whether our observations of the dependence of HFO phase synchronization on HFO amplitude (see Fig. 6) could be biased by SNR changes.

We first estimated the SEEG environmental and amplifier noise level with saline-solution recordings (Supplementary Figure 3 f). The ratio of observed signal amplitude and noise amplitude yields the apparent SNR (aSNR, Supplementary Figure 3 g). In the HFO range where the bulk of our observations were made (150-400 Hz), the aSNR values ranged from 3 to 10. We then used the numerically estimated relationship between aSNR and SNR to convert the aSNR estimates to real SNR [17]. The empirically observable ratio is A(signal+noise)/A(noise), where A indicates the amplitude operator and signal+noise indicates the sum of the real valued signal and noise time series, while SNR = A(signal)/A(noise). Using the relationship between aSNR and SNR established earlier

(see Supplementary Figure 7 of ref. 15) showed that the SNR of the SEEG HFO signals is approximately 4-10.

We then build up on previous simulation results to assess how well the true phase synchronization at various coupling levels (c) is estimated as a function of SNR [17]. The simulations show that the greatest effects of SNR on phase correlation estimates take place at SNR < 2 where the true PLV is significantly underestimated. Above that, however, the PLV estimates are very close to the asymptote level of the true PLV at SNR = inf. This already implies that given the measured SNR of 4-10 for HFO in SEEG, the SEEG PLV estimates are robust against variations in amplitude (SNR). The simulations show that improving SNR from 4 to inf will increase the PLV estimate only by approx. 10%. Now, in the measured dependence of PLV on amplitude in SEEG (see Fig. 6 a), we find that the PLV at the approx. mean amplitude (middle quintile of amplitude values) is approx. 0.06 while in the top-quintile it is >0.15. This 250% difference an order of magnitude greater than the 10% difference that one could at most expect from SNR effects. Hence, overall, SNR changes per se can be expected to play a negligible role in our HFO synchronization data.

Putative role of SEEG contact size in detecting assemblies large enough for long-range synchronization

HFOs have been observed with electrodes of greatly varying sizes, from the micro-electrodes with surface areas of 0.0013 mm^2 up to clinical macro-electrodes such as those used here (area 5 mm^2). We suggest that the larger surface area of the clinical electrodes predisposes SEEG specifically to detecting coherent spiking activity or synaptic potentials[18] that is already locally synchronized in a sizeable assembly and thus able to achieve post-synaptic impact in distant targets[19]. Hence, by construction, SEEG may be effectively filtering out both the incoherent multi-unit activity that is unlikely to achieve well-timed downstream effects as well as the post-synaptic potentials from incoherent sources.

Line-noise suppression filters did not affect phase accuracy reconstruction

In order to assess phase reconstruction accuracy, we first showed that phase dynamics were accurately reconstructed by visual inspection of the phase time-series of the original (Supplementary Figure 4 a) and filter-reconstructed signals (Supplementary Figure 4 d). Finally, we computed the PLV between the original and filter-reconstructed data for all 4 sets of oscillations from 100 to 400Hz and with 2 sampling frequencies (1kHz and 5kHz). We report that our filter approach, including the line-noise suppression FIR filters, reconstruct very accurately (PLV between true and reconstructed > 0.9) the true phase dynamics of the simulated oscillations even in presence of multiple line-noise artefacts. These results thus confirmed that the designed filter banks yielded accurate estimates of the underlying phase dynamics. Finally, to further examine whether leakage from line-noise suppression filters could possibly inflate our results we replicated the main finding (Fig. 2) using a set of FIR filter bank centered in between two line-noise harmonics and reaching the band-suppression at the line-noise harmonics. The results showed that HFO synchronization is not confounded by line-noise leakage (Supplementary Figure 4 a-f) nor from line-noise ringing (Supplementary Figure 4 g)

Phase reconstruction accuracy was not affected by near-Nyquist-limit filtering

We simulated 25 seconds of oscillatory activity embedded in uniform noise to test the phase reconstruction accuracy of our filter banks. These simulations focused mainly on the frequency range above 100Hz but *i.e.* included frequencies in the 20Hz-100Hz range for comparison purposes. As expected, the simulations yielded signals with the largest amplitude peaks at central frequency for both 5Khz and 1kHz filter-reconstructed signals (Supplementary Figure 5 c). Morlet filtering accurately reconstructed the underlying oscillations up to oscillations and resulted PLV values > 0.9 even when the simulated truth time series was down sampled from 5 kHz to 1 kHz (Supplementary

Figure 5 d). These results confirmed that our filter design approach yield high quality signals with no major phase distortions and that the reconstruction of phase dynamics was accurate even when reaching frequencies close to Nyquist limit. Thus, our observations of long-range HFO synchrony could not be explained by aliasing or poor filter design.

Differences in HFO phase synchrony between deep and superficial layers was present in bipolar data

Unlike in cWM data (see Fig .5), we found no differences in PLV between deep and superficial bipolar contacts for rhythms slower than 100Hz (Supplementary Figure 7, permutation test, $p < 0.05$ Benjamini-Hockberg corrected with α=0.05). This likely is caused by the slower oscillatory sources having size a large spatial extent that leads to cancellation or poor separability of signals from different layers in bipolar-referenced data. Oscillations above 100 Hz, however, showed different synchronization patterns between superficial and deep contacts throughout the distance ranges, as in the cWM data. This corroborates the cWM layer observations and indicates that the HFO sources are local in the meso-scale cortical circuitry.

Large-scale inter-areal synchrony in slow oscillations reflect different physiological and pathological origin

In addition to the main finding of large-scale HFO synchrony, we also observed several interesting results in frequencies below the HFO bands. First, theta-band (5–7 Hz) constituted the most pronounced large-scale phase synchrony (Fig 2 & 3). Previous studies using Granger causality have revealed that inter-areal theta-band oscillations are predominantly feedforward[20,21], and the anatomical feedforward projections originate predominantly in superficial cortical layers. Consistent with this, our data showed that long-range theta-band synchrony was stronger between superficial than between deep layers (Fig. 5; split-cohort reliability and iPLV measures see Supplementary Figure 2 & 7, respectively).

The alpha- and beta-band are two prominent rhythms in the brain [22,23]. However, we did not find pronounced peaks in those bands (Fig. 2, Fig. 3 and Fig. 5), although one may already argue the theta-peak to be an equivalent of the alpha peak in healthy EEG. There were several putative reasons for the apparent lack of prominent alpha- and beta-band synchrony. 1. Progression of chronic neurological disease is known to associate with the slowing of alpha (to theta) oscillations, e.g., in Alzheimer's disease [24] and in epilepsy with poor seizure control[25]. 2. Differences in sampling statistics could play a role: the concept of ~10 Hz alpha oscillations is driven by EEG and MEG observations in healthy subjects. These measurements are, however, biased by the greater sensitivity of EEG and MEG to superficial neocortical sources, and for MEG, overall the cortical areas closest to the MEG sensors (parietal and dorsal areas). Hence, EEG and MEG are predisposed to observing parietal and sensorimotor activities that indeed may be dominated by alpha-band oscillations. On the other hand, the sampling loci in SEEG data are often biased towards the limbic system. SEEG also samples deep cortical sources as well as superficial ones (see Fig 1 and Supplementary Figure 1 for sampling stats). In these regions, theta-band oscillations are more prevalent than alpha-band oscillations. 3. Differences in cellular generators: EEG and MEG are predisposed to observing signals from large co-oriented pyramidal neurons such as the layer 5 pyramidal neurons [26] and to lesser extent those in layer 6 neurons[27]. SEEG contacts, on the other hand, record much more local signals that are likely to include generators also in layers 4 and 2/3 neurons [4]. 4. Differences between meso- and macro-scale source architecture: intracranial recordings may reveal domains of cortical oscillations that are not evident in EEG [28]. For example, while sensory systems are characterized by alpha oscillations near 10 Hz, many other nearby brain areas exhibit lower frequencies, which may remain masked in EEG because of volume conduction and/or heterogeneity of these sources, and/or because of the second and third reasons mentioned above.

Supplementary Figures

Supplementary Figure 1

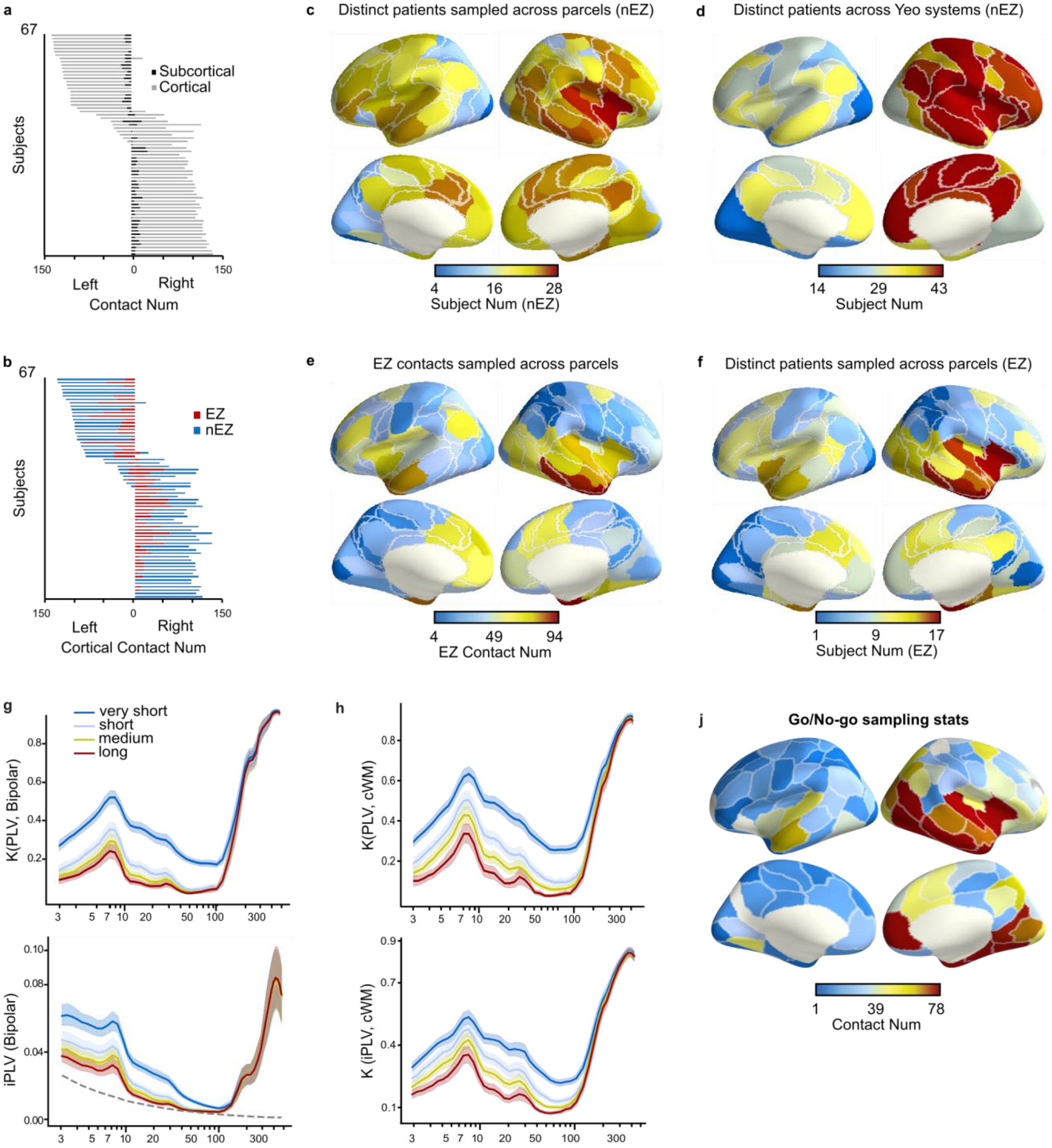

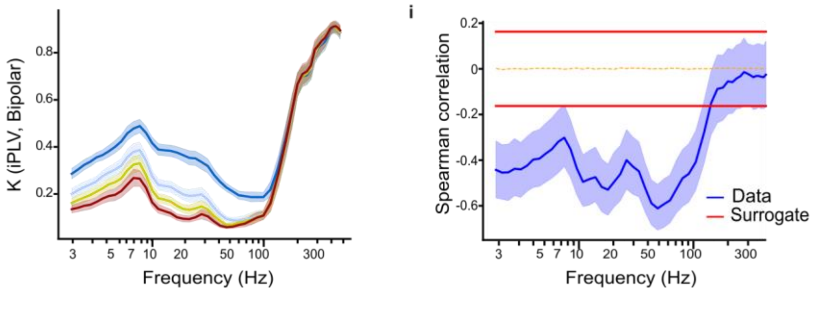

Sampling statistics and fractions of significant HFO synchrony.
a Number of SEEG contacts per subject in cortical and sub-cortical regions. **b** Numbers of epileptic (EZ) and healthy (nEZ) cortical contacts. **c** Number of subjects sampled in each Schaefer parcel using nEZ contacts only. **d** Number of subjects sampled in each of the 7 Yeo functional systems. **e** Number of distinct EZ contacts across subjects for each of 100 cortical parcels. **f** Number of subjects with at least one EZ contact in each of 100 cortical parcels.
g–h Supporting data for Fig. 2**a–c**. *K*: connection density of significant PLV or iPLV in contact pairs (one-sided test; $p < 0.001$ for observed > surrogate). Thick lines are the mean and shaded areas represent confidence limits (two-tail; $p < 0.05$) derived from 100 bootstraps. Dashed lines are surrogate (N = 100) data level for $p < 0.001$. **i** Spearman correlation coefficient between distance bins and corresponding PLV value. Shaded areas are confidence limits derived from 1000 bootstraps**. j** Number of cortical contacts in the Go/no-Go cohort.

Supplementary Figure 2

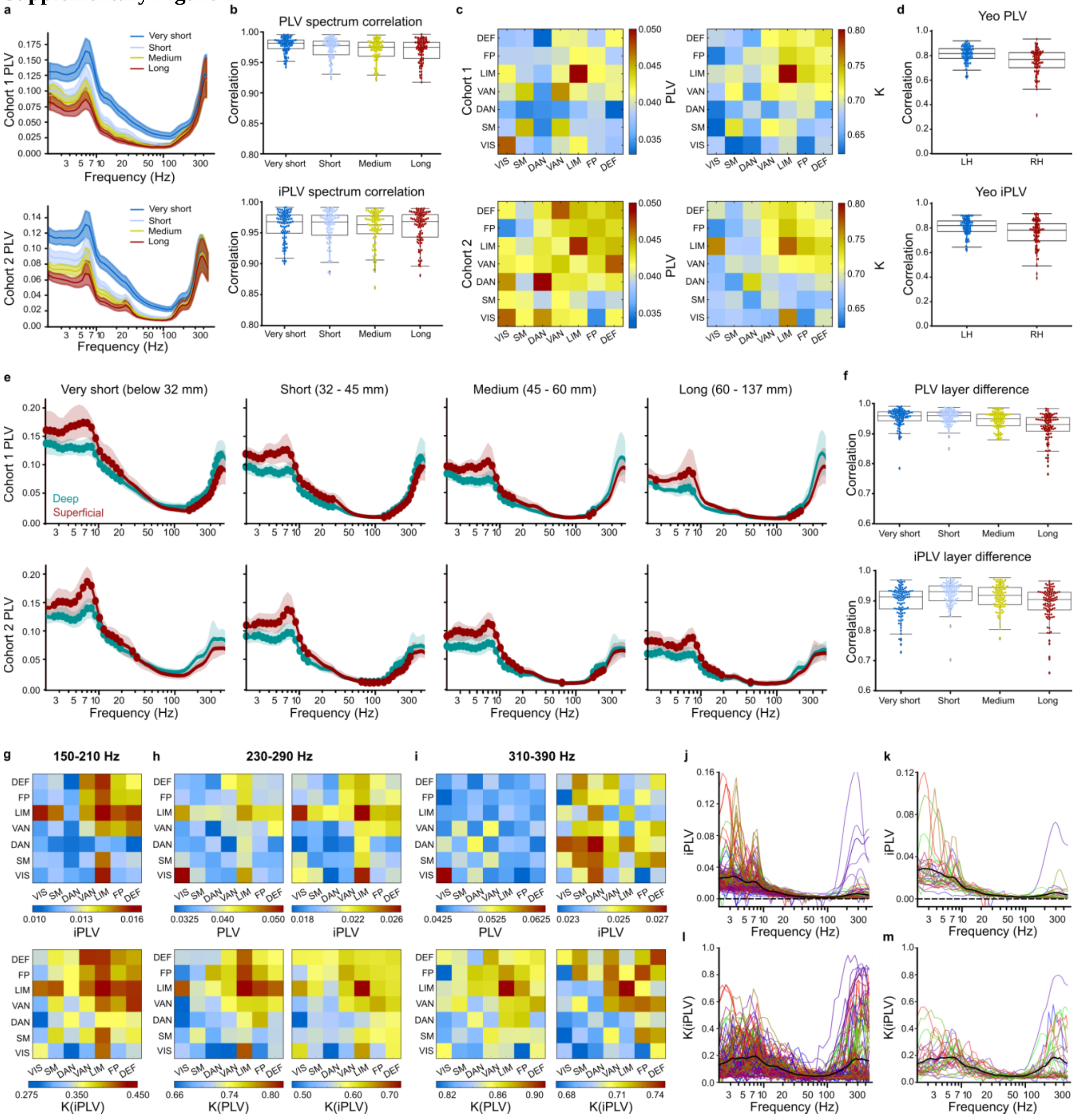

Replicating HFO synchrony profiles in split-cohort and an external dataset.

a Large-scale phase synchrony profiles across distances for the split-cohort with maximal spatial correlation. Shaded areas are confidence limits (two-tail; $p < 0.05$) derived from 100 bootstraps. **b** Correlation between single-subject average PLV cohorts (N_1=33, N_2=33) of the 100 random split-cohort solutions (dots) to the full-cohort observation (Fig 2). **c** Example of split-cohort observation of 150-210 Hz synchrony between Yeo systems. **d** Correlation of the random (N=100) split-cohort solutions (dots) to the full-cohort observation (Fig 4). **e** Example of split-cohort observation of PLV layer profiles; red: superficial, teal: deep. Shaded areas are confidence limits (two-tail; $p < 0.05$) derived from 100 bootstraps. **f** Correlation of the 100 random split-cohort solutions (N_1=33, N_2=33) (dots) to the full-cohort observation (Fig 5). **g** Phase synchronization strengths (PLV and iPLV) and extent (K) between Yeo systems for 150-210Hz, **h** 230-290Hz, and **i** 310-390Hz estimated from subject cluster 1 and 2, *i.e.*, used for community detection (see Fig 4). **j** Individual and mean difference of iPLV and corresponding surrogate spectrums for each of the 138 available recordings and **k** for each recording conditions (N=13) average within subjects (N=31). Recordings from the same subjects are indicated with the same color. Thick black line represents the average across all recordings and all subjects. Dashed black line represents surrogate level. **l** Fraction of significant iPLV edges for each recording, and **m** for each conditions average within subjects. Different subjects are indicated with different color. All boxes in this figure indicate inter-quartile range (IQR), markers in the box indicate median, distances between whiskers indicate 1.5*IQR.

Supplementary Figure 3

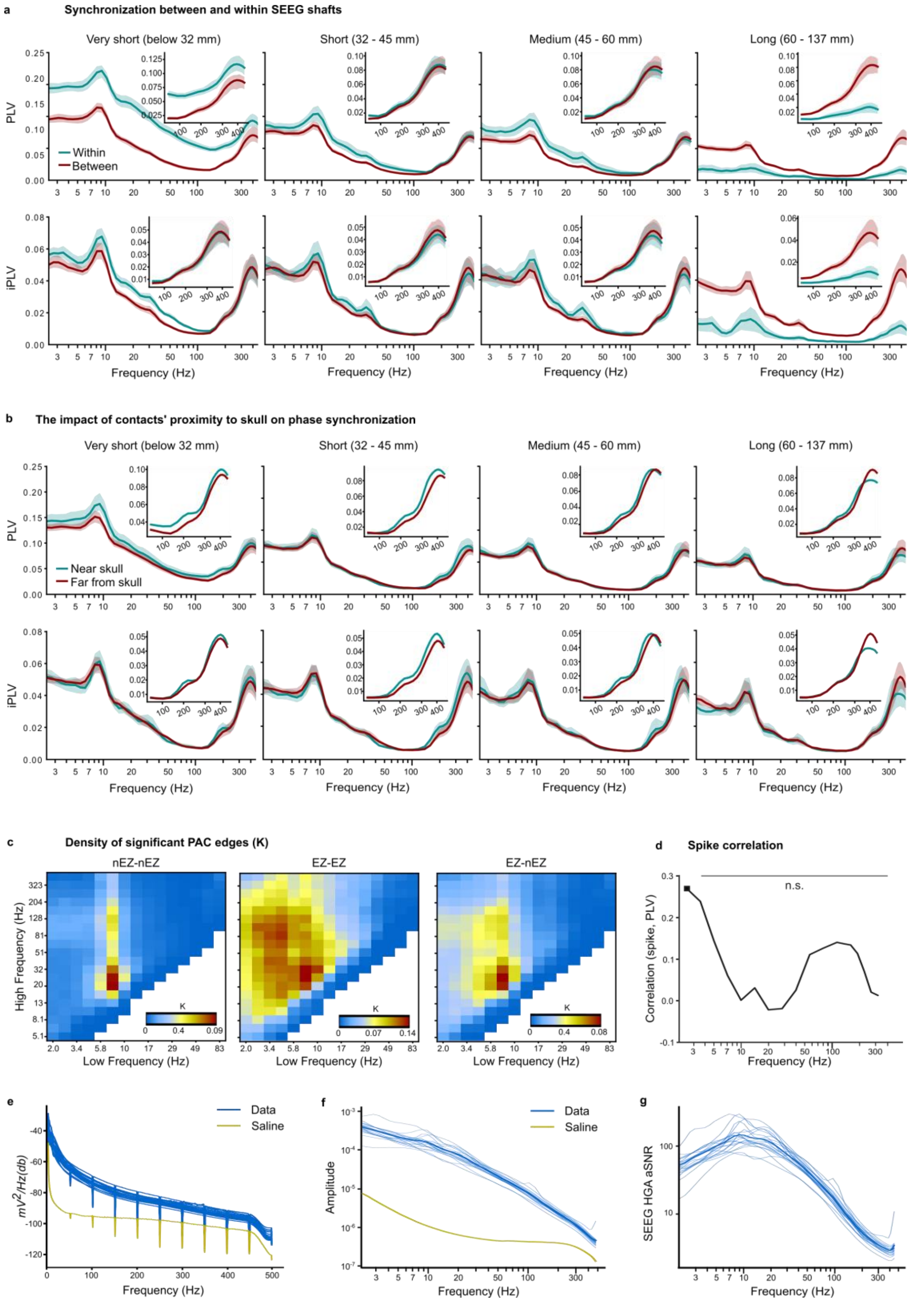

Neither artefacts nor pathology confounded HFO phase synchrony.
a Average (thick lines) PLV and iPLV between contacts from different shafts (red) and the same shaft (azure) for all distance ranges. Shaded areas represent confidence limits (two-tail; $p < 0.05$) from bootstrapped (N = 100) of population variance. **b** Average (thick lines) PLV and iPLV between near-skull (red) and far-from-skull (azure) contacts as a function of frequency for all distance ranges. Shaded areas represent confidence limits. **c** Connection density of PAC PLV edges shown in Fig 7a. **d** Pearson correlation coefficient between PLV and frequency of inter-ictal events across frequencies. Square marker: significant correlation ($p < 0.05$, uncorrected)**. e** Comparison of Power Spectral Density, **f** signal amplitude, and **g** apparent Signal-to-noise ratio (aSNR) between real cortical signals (N=18) and saline solution. Thin lines represent individual subjects, thick line represents the mean across subjects.

Supplementary Figure 4

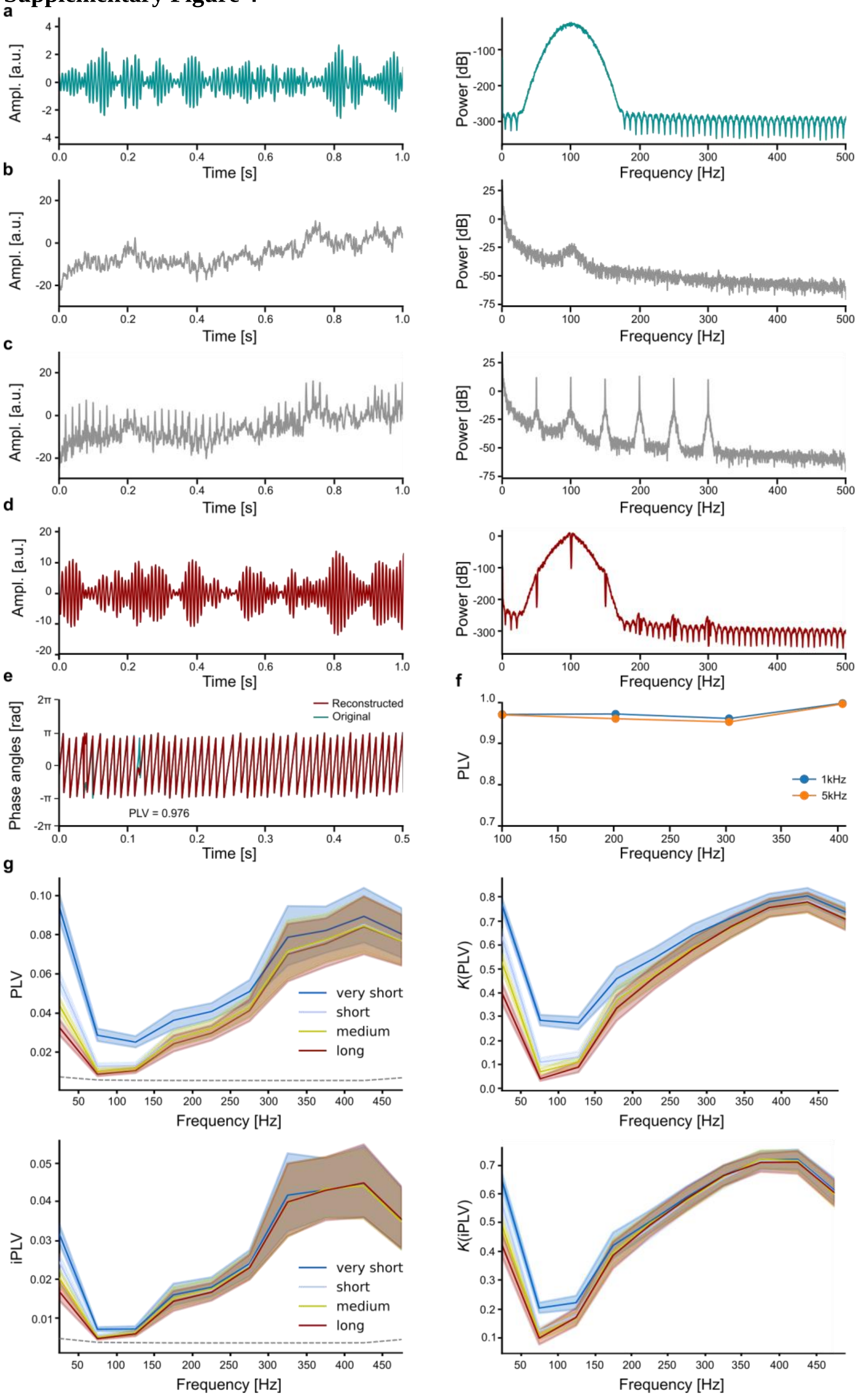

Line-noise suppression filters or line-noise leakage do not affect phase estimation accuracy.
a Time course of ground-truth 100Hz oscillation, **b** with 1/f additive noise, and **c** with line-noise harmonics and their power spectrums. **d** Filter-reconstructed 100Hz oscillation and its power spectrum. **e** Phase time-series of original (blue) and filter-reconstructed (red) 100Hz oscillations. **f** time-averaged phase difference (PLV) across 4 different oscillations from 100Hz to 400Hz for 1kHz (blue) and 5kHz (orange) sampled data. **g** Phase synchrony strength (PLV/iPLV) and extent (*K*) for cWM referenced data using FIR band-pass filters centered in between two line-noise harmonics.

Supplementary Figure 5

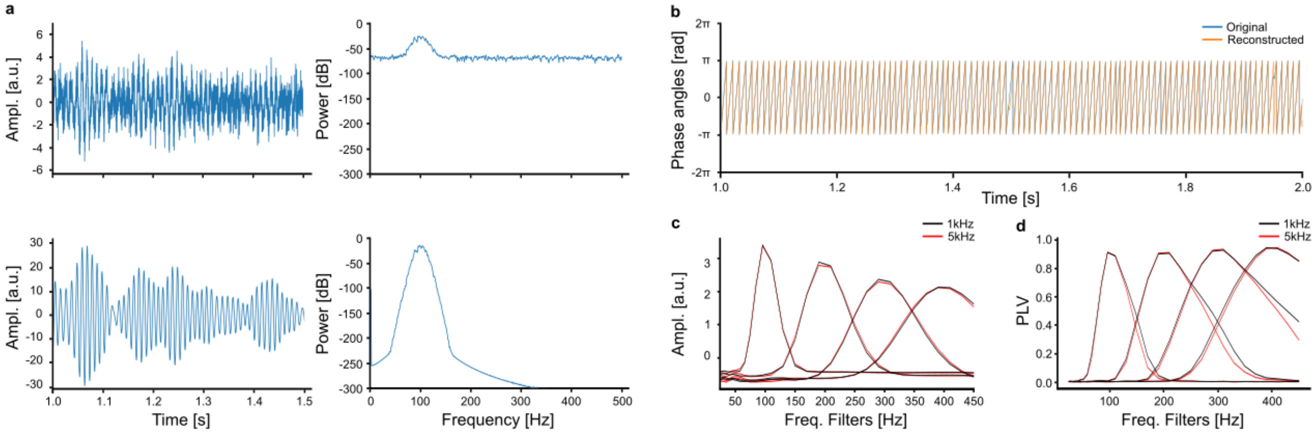

Phase reconstruction accuracy was not affected by filtering near the Nyquist limit.
a A randomly selected segment of simulated original truth time series (100Hz) with additive uniform noise (top) and filter-reconstructed oscillation trace (bottom) along with their relative spectrums (right columns). **b** Phase of the original oscillation (blue) and filter-reconstructed oscillation (orange). **c** Average amplitude profiles and **d** phase differences for 4 simulated oscillations from 100Hz to 400 Hz for 1kHz (black) and 5kHz (red) sampling rate.

Supplementary Figure 6

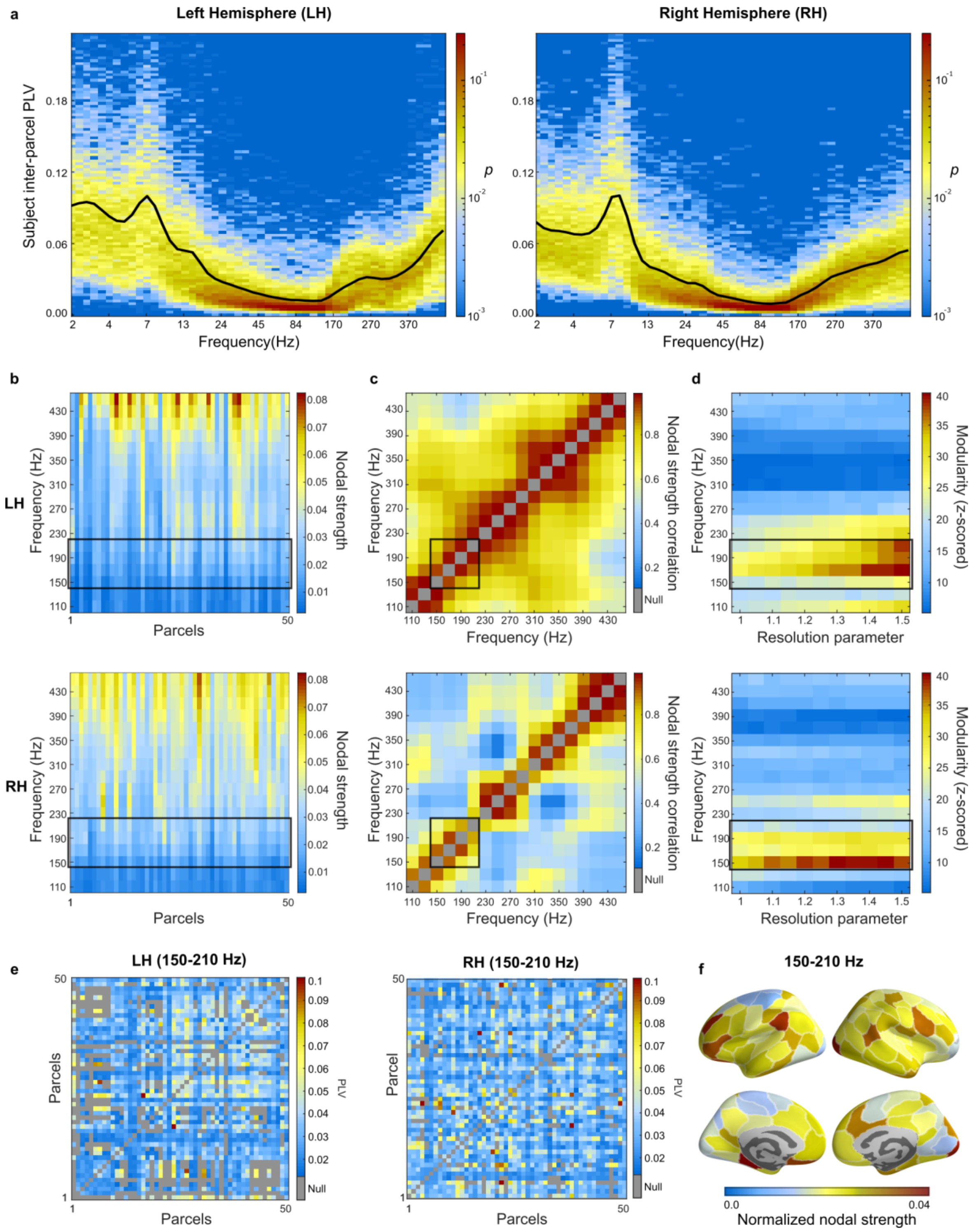

Community structure in HFO synchrony networks

a Probability (*p*) distribution of narrow-band inter-parcel PLV (50 Schaefer parcels per hemisphere). Overlaid lines are the narrow-band mean inter-parcel PLV across frequencies. **b** Nodal strength of Schaefer parcels, **c** correlation of nodal strength across HFO frequencies, **d** normalized (z-score) modularity as a function of the resolution parameter for community detection in narrow-band HFO PLV networks, for both left (top) and right hemispheres (bottom). Black boxes demarcate the HFO band of interest (150-210 Hz). **d** Inter-parcel PLV in all HFO frequencies and γ combinations showed significant modularity (permutation test, $p<0.05$, N=100, one-tailed). **e** Collapsed 150-210 Hz inter-parcel PLV matrices for left and right hemispheres. Grey values indicate unassigned parcel-pairs. **f** Cortical maps of nodal strength for 150-210 Hz inter-parcel PLV matrices.

Supplementary Figure 7

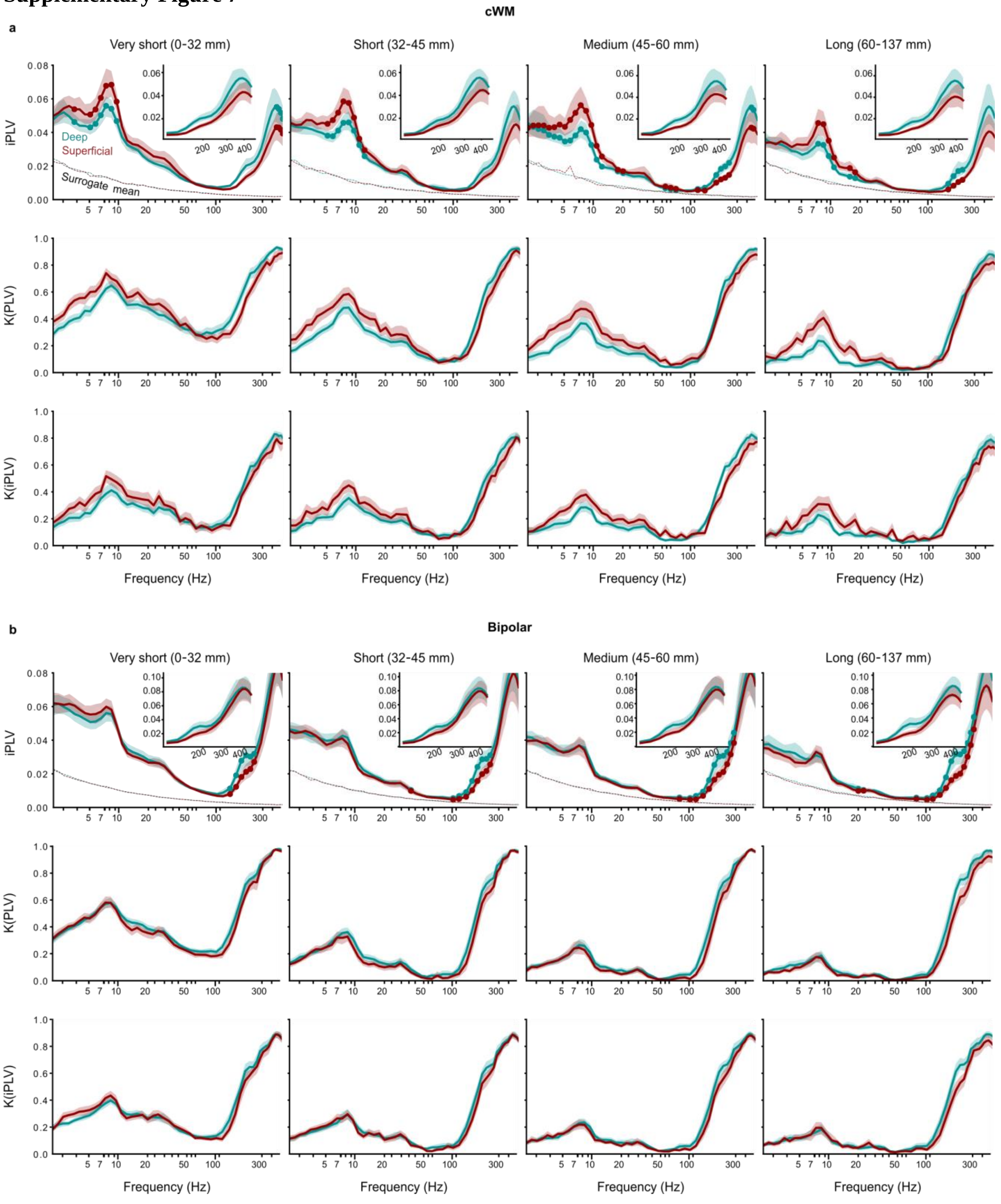

Distinct layers show different synchrony profiles.
a Spectrum profiles of phase synchronization (iPLV) between distinct layer profiles for cWM and **b** bipolar referenced data (both PLV and iPLV). K indicates connection density of significant PLV and iPLV. Thick lines represent mean and shaded areas represent 2.5% and 97.5% confidence limits for bootstrapped values (N = 100). Markers represent significance for a two-tail permutation test (N = 100) over edges with $p < 0.05$.

ID	EZ Location	Age	Resting state Duration	Sex	Drugs	Outcome (Engel score)
1	Right mesial frontal	21	15:01	M	Carbamazepine 600mg, Levetiracetam 1500mg	IB (8 years)
2	Right temporal insular	26	9:53	M	Carbamazepine 1200mg, Primidone 750 mg, Clonazepam 10mg	IA (37 months)
3	Right temporal	38	10:29	M	Carbamazepine 1200mg, Dilantin 450mg, Lacosamide 300mg, Clonazepam 10mg	No surgery
4	Left temporo-parietal	38	10:51	F	Phenobarbital 100mg, Topiramate 100mg, Levetiracetam 3000mg	IA (25 months)
5	Left temporal-insular	24	10:48	M	Oxcarbazepine 600mg, Lacosamide 400mg	IA (15 months)
6	Right temporo-insular	40	10:33	F	Levetiracetam 1000mg, Lacosamide 350mg, Sertraline 50mg, Lorazepam 1mg	IIIA (32 months)
7	Left temporal- orbital*	38	11:22	M	Carbamazepine 1200 mg, Levetiracetam 3500mg, Topiramate 200mg	IA (3 years)
8	Precuneus	19	11:30	M	Oxcarbazepine 600mg, Lacosamide 400mg	No surgery
9	Functional epilepsy	20	13:13	F	Carbamazepine 1200mg, Levetiracetam 1500mg	No surgery
10	Right occipito-temporo-parietal	20	10:23	M	Lamotrigine 400mg, Levetiracetam 3000mg, Lacosamide 400mg	IA (3 years)
11	Right temporo-occipital	41	10:03	M	Phenytoin 200mg, Lacosamide 300mg	IA (6 months)
12	Left temporal	35	10:05	M	Topiramate 200mg, Carbamazepine 900mg	IA (12 months)
13	Right temporal anterior	28	10:23	M	Carbamazepine 1200mg	IIA (66 months)
14	Temporo-hip	36	11:26	F	Carbamazepine 1400mg, Levetiracetam 3000mg	IIA (6 months)
15	Left frontal anterior	40	10:01	M	Levetiracetam 2750mg, Carbamazepine 800mg, Primidone 750mg	IA (12 months)
16	Thermo-coagulation multiple sites	39	9:58	M	Oxcarbazepine 1800mg, Clobazam 20mg	IA (36 months)
17	Right fronto-temporo-insular	24	10:03	F	Carbamazepine 1000mg, Clobazam 20mg, Lomotrigine 200mg	IVA (24 months)
18	Left parieto-opercolo-insular	31	11:09	F	Carbamazepine 1200mg, Clobazam 40mg, Phenobarbital 75mg	IVA (12 months)
19	Right temporo-perisilvian	34	10:02	F	Phenobarbital 150mg, Lacosamide 400mg, Clobazam 10mg	IIC (38 months)
20	Right perisilvian-insular	17	10:36	M	Carbamazepine 800 mg, Lamotrigine 400mg	IVA (26 months)
21	Right temporo-parieto-occipital	36	10:09	M	Oxcarbazepine 1200mg, Phenobarbital 150mg, Valproate 1000mg	IA (35 months)
22	--	32	14:40	F	Carbamazepine 700mg	No surgery
23	Left temporal antero-mesial	32	11:26	M	Carbamazepine 1200mg, Levetiracetam 750mg	IA (61 months)
24	Right fronto-centro-insular	33	10:11	M	Carbamazepine 800mg, Lacosamide 800mg, Zonisamide 250mg	IIA (38 months)
25	Left temporal	21	10:07	F	Levetiracetam 1750mg, Lacosamide 400mg, Valproate1000mg	IA (24 months)
26	Right parietal	23	10:54	M	Levetiracetam 3000mg, CBZ 1000mg, Lacosamide 500mg	IA (24 months)
27	Thermo-coagulation	46	10:02	M	Carbamazepine 1200mg, Phenobarbital 100mg	IA (24 months)
28	Right frontal	20	10:48	F	Valproate 800mg, Clobazam 10mg	IIA (36 months)
29	Right fronto-mesial	21	16:55	M	Carbamazepine 800mg, Levetiracetam 3000mg, Nitrazepam 1.5mg	IIIA (13 months)
30	Right fronto-central	22	10:10	M	Lamotrigine 400mg, Levetiracetam 2000mg	IA (24 months)
31	Right frontal	20	10:33	M	Carbamazepine 600mg, Rufinamide 1500mg	IVA (13 months)
32	Right frontal	44	10:05	F	Carbamazepine 1200mg, Zonisamide 400mg, Phenobarbital 1000mg	IC (24 months)
33	--	17	11:13	M	Carbamazepine 300mg	No surgery
34	--	14	10:05	M	Levetiracetam 1500 mg, Clobazam 5mg	No surgery
35	Right temporal antero-mesial	30	10:19	F	Oxcarbazepine 2000mg, Phenobarbital 150mg	IIA (36 months)
36	--	24	10:01	M	Carbamazepine 16000mg, Levetiracetam 4000mg	No surgery
37	--	29	9:53	F	Levetiracetam 3000mg	No surgery
38	Right orbito-temporal	29	9:56	F	Zonisamide 400mg, Levetiracetam 750mg, Carbamazepine 1400mg	IA (62 months)
39	--	45	10:10	F	Lacosamide 500mg, Valproate 1000mg, Zonisamide 200mg	No surgery
40	Thermo-coagulation multiple sites	34	10:00	F	Carbamazepine 1000mg, Levetiracetam 2500mg	IA (12 months)
41	Thermo-coagulation multiple sites	50	10:00	M	Levetiracetam 2000mg, Lacosamide 600mg	IIA (6 months)
42	Left occipital	17	10:03	F	Carbamazepine 1200mg, Levetiracetam 1500mg, Lacosamide 300mg	IB (49 months)
43	Right temporal	44	10:03	F	Topiramate 300mg, Oxcarbamazepine 1200mg	IIA (50 months)

44	--	27	9:58	M	Carbamazepine 800mg, Lamotrigine 200mg	No surgery
45	--	46	10:01	M	Carbamazepine 1200mg, Levetiracetam 3000mg, Lacosamide 150mg, Clobazam 20mg	No surgery
46	Left cingulum	30	10:02	M	Oxcarbamazepine 1800mg, Topiramate 200mg, Levetiracetam 3000mg, Clobazam 10mg	IA (16 months)
47	Right antero-frontal	28	11:07	M	Carbamazepine 1000mg, Levetiracetam 1000mg	IIIA (61 months)
48	Thermo-coagulation right temporo-parieto-perisilvian	27	10:01	F	Topiramate 200mg, Lamotrigine 200mg	IA (5 years)
49	Right temporal antero-mesial	42	11:28	F	Lacosamide 500mg	IB (36 months)
50	Left parieto-temporal	15	10:08	M	Carbamazepine 900mg	IA (6 months)
51	Thermo-coagulation right temporo-opercular	37	10:02	M	Carbamazepine 900mg, Levetiracetam 3000mg	IVA (12 months)
52	Left frontal	30	10:16	F	Carbamazepine 1200mg, Lamotrigine 200mg, Clobazam 20mg	IA (5 years)
53	Left frontal	15	10:00	F	Levetiracetam 1250mg, Oxcarbamazepine 1200mg	IA (4 years)
54	Thermo-coagulation	41	10:04	M	Levetiracetam 3000mg, Lacosamide 400mg	IA (2 years)
55	Right temporo-occipital	37	10:07	M	Lamotrigine 600mg, Levetiracetam 2000mg	IA (2 years)
56	Right temporal	29	10:20	M	Carbamazepine 1400mg, Levetiracetam 3000mg, Clobazam 10mg	IA (31 months)
57	Left opercolo-insular	10	10:02	F	Carbamazepine 800mg	IIIA (34 months)
58	Right temporo-frontal	40	10:02	F	Lamotrigine 600mg, Clobazam 20mg, Phenytoin 500mg	IA (13 months)
59	Left temporo-insular-operculum	29	11:04	F	Carbamazepine 1800mg, Clobazam 20mg	IA (24 months)
60	Right temporal	27	11:15	M	Lamotrigine 400mg, Topiramate 400mg	IA (24 months)
61	--	26	9:54	M	Phenytoin 400mg, Topiramate 500mg	No surgery
62	Left temporal	17	10:15	M	Oxcarbamazepine 1500mg, Clobazam 20mg, Levetiracetam 2500mg	IA (36 months)
63	Right temporo-mesial	25	11:03	F	Topiramate 75mg, Carbamazepine 1500mg	IA (12 months)
64	Nodular heterotopia	24	10:11	F	Carbamazepine 1000mg, Levetiracetam 500mg, Clobazam 20mg	IVA (12 months)
65	Left temporo-perisilvian	37	9:54	F	Carbamazepine 1200mg, Lamotrigine 550mg	IIIA (12 months)
66	Left temporal antero-mesial	32	10:30	F	Clobazam 20mg, Phenobarbital 45mg	IIA (55 months)
67	Right temporo-occipital	44	10:02	M	Carbamazepine 800mg, Levetiracetam 3000mg, Phenobarbital 125mg	IA (24 months)

Supplementary Table 1 Demographic data for main cohort. EZ location refers to the supposed brain location of the epileptogenic zone. Thermo-coagulation refers to the practice used in drug-resistant focal epilepsy where current is injected in a bipolar derivation in order to increase the peri-contact temperature. Dashed entries in EZ locations mean that no single focal location was identified. Age column represent the age of the patients at the recording date. Durations are expressed in minutes. Drugs are reported with their active principle names. Drug dosage is expressed milligrams and refers to the morning dosage measured at the day of the recording. Outcome is expressed as Engel scores and number in parenthesis refer to the point time after surgery when the visit occurred.

Supplementary References

1. Palva, J. M. *et al.* Ghost interactions in MEG/EEG source space: A note of caution on inter-areal coupling measures. *Neuroimage* 173, 632–643 (2018).
2. Palva, S. & Palva, J. M. Discovering oscillatory interaction networks with M/EEG: Challenges and breakthroughs. *Trends in Cognitive Sciences* vol. 16 219–230 (2012).
3. Vinck, M., Oostenveld, R., Van Wingerden, M., Battaglia, F. & Pennartz, C. M. A. An improved index of phase-synchronization for electrophysiological data in the presence of volume-conduction, noise and sample-size bias. *Neuroimage* (2011).
4. Arnulfo, G., Hirvonen, J., Nobili, L., Palva, S. & Palva, J. M. Phase and amplitude correlations in resting-state activity in human stereotactical EEG recordings. *Neuroimage* 114–127 (2015).
5. Arnulfo, G., Narizzano, M., Cardinale, F., Fato, M. M. & Palva, J. M. Automatic segmentation of deep intracerebral electrodes in computed tomography scans. *BMC Bioinformatics* 16: 99 (2015).
6. Traag, V., Waltman, L. & van Eck, N. J. From Louvain to Leiden: guaranteeing well-connected communities. (2018).
7. Williams, N. *et al.* Comparison of methods to identify modules in noisy or incomplete brain networks. *Brain Connect.* 9, 128–143 (2019).
8. Miller, K. J., Sorensen, L. B., Ojemann, J. G. & Den Nijs, M. Power-law scaling in the brain surface electric potential. *PLoS Comput. Biol.* 5, (2009).
9. Miller, K. J. A library of human electrocorticographic data and analyses. *Nat. Hum. Behav.* 3, 1225–1235 (2019).
10. Otsubo, H. *et al.* High-frequency oscillations of ictal muscle activity and epileptogenic discharges on intracranial EEG in a temporal lobe epilepsy patient. *Clin. Neurophysiol.* 119, 862–8 (2008).
11. Palva, S. & Palva, J. M. Discovering oscillatory interaction networks with M/EEG: Challenges and breakthroughs. *Trends Cogn. Sci.* 16, 219–30 (2012).
12. Baumann, S., Wozny, D., Kelly, S. & Meno, F. The Electrical Conductivity of Human Cerebrospinal Fluid at Body Temperature. *IEEE Trans. Biomed. Eng.* 44, (1997).
13. Cardinale, F. *et al.* Stereoelectroencephalography: Retrospective analysis of 742 procedures in a single centre. *Brain* 142, 2688–2704 (2019).
14. Cardinale, F. *et al.* Stereoelectroencephalography: Surgical methodology, safety, and stereotactic application accuracy in 500 procedures. *Neurosurgery* (2013).
15. Cardinale, F. *et al.* A new tool for touch-free patient registration for robot-assisted intracranial

surgery: Application accuracy from a phantom study and a retrospective surgical series. *Neurosurg. Focus* 42, (2017).

16. Lhatoo, S., Kahane, P. & Lüders, H. *Invasive Studies of the Human Epileptic Brain: Principles and Practice*. Oxford University Press, (2018).

17. Palva, J. M., Monto, S., Kulashekhar, S. & Palva, S. Neuronal synchrony reveals working memory networks and predicts individual memory capacity. *Proc. Natl. Acad. Sci. U. S. A.* 107, 7580–7585 (2010).

18. Châtillon, C. E. *et al.* Influence of contact size on the detection of HFOs in human intracerebral EEG recordings. *Clin. Neurophysiol.* 124, 1541–6 (2013).

19. Singer, W. Neuronal Synchrony: A Versatile Code for the Definition of Relations? *Neuron* 24, 49–65 (1999).

20. Bastos, A. M. *et al.* Visual areas exert feedforward and feedback influences through distinct frequency channels. *Neuron* 85, 390–401 (2015).

21. Spyropoulos, G., Bosman, C. A. & Fries, P. A theta rhythm in macaque visual cortex and its attentional modulation. *Proc. Natl. Acad. Sci. U. S. A.* 115, E5614–E5623 (2018).

22. Buzsáki, G., Anastassiou, C. A. & Koch, C. The origin of extracellular fields and currents-EEG, ECoG, LFP and spikes. *Nat. Rev. Neurosci.* 407–420 (2012).

23. Buzsáki, G. & Draguhn, A. Neuronal Oscillations in Cortical Networks. *Science (80-.).* 304, 1926 (2004).

24. de Frutos-Lucas, J. *et al.* Physical activity effects on the individual alpha peak frequency of older adults with and without genetic risk factors for Alzheimer's Disease: A MEG study. *Clin. Neurophysiol.* 129, 1981–1989 (2018).

25. Abela, E. *et al.* Slower alpha rhythm associates with poorer seizure control in epilepsy. *Ann. Clin. Transl. Neurol.* 6, 333–343 (2019).

26. Jones, S. R., Pritchett, D. L., Stufflebeam, S. M., Hämäläinen, M. & Moore, C. I. Neural correlates of tactile detection: A combined magnetoencephalography and biophysically based computational modeling study. *J. Neurosci.* 27, 10751–10764 (2007).

27. Lee, S. & Jones, S. R. Distinguishing mechanisms of gamma frequency oscillations in human current source signals using a computational model of a laminar neocortical network. *Front. Hum. Neurosci.* 7, (2013).

28. Frauscher, B. *et al.* Atlas of the normal intracranial electroencephalogram: Neurophysiological awake activity in different cortical areas. *Brain* 141, 1130–1144 (2018).
