# Supplementary material for: Long-range phase synchronization of high-frequency oscillations in human cortex
Source: Nat Commun. 2020 Oct 23;11:5363. doi: 10.1038/s41467-020-18975-8 (PMC7584610; doi:10.1038/s41467-020-18975-8)
Supplement: Supplementary file 2 — Reporting Summary [file 41467_2020_18975_MOESM2_ESM.pdf]

nature research

Corresponding author(s): J. Matias Palva, Gabriele Arnulfo

Last updated by author(s): Jul 11, 2020

Reporting Summary

Nature Research wishes to improve the reproducibility of the work that we publish. This form provides structure for consistency and transparency in reporting. For further information on Nature Research policies, see our Editorial Policies and the Editorial Policy Checklist.

Statistics

For all statistical analyses, confirm that the following items are present in the figure legend, table legend, main text, or Methods section.

n/a	Confirmed	
☐	☒	The exact sample size (*n*) for each experimental group/condition, given as a discrete number and unit of measurement
☐	☒	A statement on whether measurements were taken from distinct samples or whether the same sample was measured repeatedly
☐	☒	The statistical test(s) used AND whether they are one- or two-sided<br>*Only common tests should be described solely by name; describe more complex techniques in the Methods section.*
☒	☐	A description of all covariates tested
☐	☒	A description of any assumptions or corrections, such as tests of normality and adjustment for multiple comparisons
☐	☒	A full description of the statistical parameters including central tendency (e.g. means) or other basic estimates (e.g. regression coefficient) AND variation (e.g. standard deviation) or associated estimates of uncertainty (e.g. confidence intervals)
☐	☒	For null hypothesis testing, the test statistic (e.g. F, t, r) with confidence intervals, effect sizes, degrees of freedom and P value noted<br>*Give P values as exact values whenever suitable.*
☒	☐	For Bayesian analysis, information on the choice of priors and Markov chain Monte Carlo settings
☒	☐	For hierarchical and complex designs, identification of the appropriate level for tests and full reporting of outcomes
☒	☐	Estimates of effect sizes (e.g. Cohen's *d*, Pearson's *r*), indicating how they were calculated

Our web collection on statistics for biologists contains articles on many of the points above.

Software and code

Policy information about availability of computer code

Data collection	SEEG data have been collected using Nihon Kohden acquisition software (v1100A, 1200A). MRI images have been acquired using Philips Achieva 1.5T scanner. CT images have been acquired with Medtronic O-Arm scanner.
Data analysis	Data analysis have been conducted using custom software written in Python. Cortical surfaces have been using Freesurfer (6.0.0) from single-subject T1 MRI data. All code supporting our results can be found at https://github.com/palvalab/seeg_hfo_synchronization/

For manuscripts utilizing custom algorithms or software that are central to the research but not yet described in published literature, software must be made available to editors and reviewers. We strongly encourage code deposition in a community repository (e.g. GitHub). See the Nature Research guidelines for submitting code & software for further information.

Data

Policy information about availability of data

All manuscripts must include a data availability statement. This statement should provide the following information, where applicable:

- Accession codes, unique identifiers, or web links for publicly available datasets
- A list of figures that have associated raw data
- A description of any restrictions on data availability

Raw data and patient details cannot be shared due to Italian governing laws as well as Ethical committee restrictions. Intermediate as well as final processed data that support the findings of this study are available from the corresponding author upon reasonable request.

Field-specific reporting

Please select the one below that is the best fit for your research. If you are not sure, read the appropriate sections before making your selection.

☒ Life sciences ☐ Behavioural & social sciences ☐ Ecological, evolutionary & environmental sciences

For a reference copy of the document with all sections, see nature.com/documents/nr-reporting-summary-flat.pdf

Life sciences study design

All studies must disclose on these points even when the disclosure is negative.

Sample size	We selected as many subjects as possible to reach at least 80% of cortical coverage (see Arnulfo et al. 2015 and Fig.1 in current MS).
Data exclusions	Exclusion criteria are indicated in the main text. Briefly we excluded subjects that have undergone to previous neurosurgical intervention or those who had significant brain injuries (e.g. tumors) that might impair some analysis (e.g. brain surface extraction). We also discarded defective electrodes that were clearly recording artefactual signals. Exclusion criteria were merely linked to major structural malformation and were not pre-estabilished.
Replication	We replicated all major results with two different approaches. We used a split cohort approach (N=100 random splits) as well as applied our analyses pipeline to an independent open-access database of ECoG data. All replication attempts were successful as reported in relevant section of the paper and figures
Randomization	There are no groups of patients in this study in main results. Patients have been divided in groups only relation to one specific analysis and the procedure has been extensively described in the main text.
Blinding	There are no groups of patients in this study in main results.

Reporting for specific materials, systems and methods

We require information from authors about some types of materials, experimental systems and methods used in many studies. Here, indicate whether each material, system or method listed is relevant to your study. If you are not sure if a list item applies to your research, read the appropriate section before selecting a response.

Materials & experimental systems

n/a	Involved in the study	
☒	☐	Antibodies
☒	☐	Eukaryotic cell lines
☒	☐	Palaeontology and archaeology
☒	☐	Animals and other organisms
☐	☒	Human research participants
☒	☐	Clinical data
☒	☐	Dual use research of concern

Methods

n/a	Involved in the study	
☒	☐	ChIP-seq
☒	☐	Flow cytometry
☒	☐	MRI-based neuroimaging

Human research participants

Policy information about studies involving human research participants

Population characteristics	In the present study we analyzed drug-resistant focal epileptic patients undergoing pre-surgical evaluations. Age, sex, and drug-level distribution across subjects are reported in Supplementary Table 1 in the main manuscript.
Recruitment	Patients have been recruited at the Niguarda Hospital of Milan progressively without any restrictions. Exclusion criteria are indicated in the main text. Briefly we excluded subjects that have undergone to previous neurosurgical intervention or those who had significant brain injuries (e.g. tumors) that might impair some analysis (e.g. brain surface extraction). There was no selection bias, we recruited all patients coming to the center independently on other factors .
Ethics oversight	The study was conducted following the Declaration of Helsinki, patients gave their informed consent and the study was approved by local Ethical Committee of Niguarda Hospital (ID 939)

Note that full information on the approval of the study protocol must also be provided in the manuscript.
